# Pharmacokinetics and brain tissue distribution of *Gastrodia elata* extract in normal and cerebral ischemic rats: a comparative study

**DOI:** 10.3389/fphar.2025.1624576

**Published:** 2025-07-31

**Authors:** Ji Shao, Qi Xu, Yunyun Luo, Ting Dong, Xue Han, Bilian Chen, Huazhong Ying, Cuifen Fang, Qiaojuan Shi

**Affiliations:** ^1^Zhejiang Provincial Key Laboratory of Laboratory Animals and Safety Research, Hangzhou Medical College, Hangzhou, China; ^2^NMPA Key Laboratory of Quality Evaluation of Traditional Chinese Medicine, Zhejiang Institute for Food and Drug Control, Hangzhou, China

**Keywords:** Gastrodia elata, cerebral ischemia, pharmacokinetics, brain tissue distribution, xenobiotic metabolite identification

## Abstract

**Objective:**

This study systematically investigated the pharmacokinetic characteristics, cerebral distribution, and metabolic transformation of *Gastrodia elata* components in both healthy and cerebral ischemia rat models.

**Methods:**

Chemical profiling of *Gastrodia elata* was conducted using UPLC-Q-TOF-MS. Based on the systemically absorbed constituents identified in plasma and brain tissues of dosed rats, a validated UPLC-QQQ-MS method was established to quantitatively determine target compound levels in plasma and brain tissues of both normal and cerebral ischemic rats following 3-day oral administration. Subsequently, UPLC-Q-TOF-MS was reapplied to conduct identification and comparative analysis of xenobiotic metabolites in both *in vivo* systems and brain tissues.

**Results:**

Based on the established therapeutic efficacy of *Gastrodia elata* extract against cerebral ischemia-reperfusion injury, chemical profiling identified 53 constituents, among which six were simultaneously detected in plasma and brain tissue of dosed rats. The established simultaneous quantitative analytical method demonstrated reduced gastrointestinal absorption of parishin A, parishin B, parishin C, parishin E and gastrodin in ischemic model rats compared to healthy controls. Notably, brain accumulation of these compounds was significantly increased in ischemic models, attributable to compromised blood-brain barrier integrity. Xenobiotic metabolite analysis identified nine biotransformation products, four of which exhibited quantifiable exposure levels in brain tissues across all experimental groups.

**Conclusion:**

This study systematically revealed the bioactive components of *Gastrodia elata* and their cerebral distribution patterns. The pharmacokinetic characteristics of gastrodin and related bioactive compounds were also elucidated. These findings provide a valuable reference for pharmacological exploration, safety evaluation, and clinical application of *Gastrodia elata* in cerebrovascular disorders.

## 1 Introduction

Stroke ranks as the second leading global cause of mortality (6.6 million annual deaths) and the third primary contributor to disability worldwide (Feigin et al., 2023). Ischemic stroke, representing over 80% of cases, arises from cerebral hypoperfusion due to vascular occlusion, culminating in ischemic necrosis and neurological deficits. While timely reperfusion remains the cornerstone therapeutic intervention, paradoxical reperfusion injury frequently manifests as blood-brain barrier (BBB) disruption, cerebral edema, and exacerbated neuronal damage (Guo et al., 2016), critically impacting patient prognosis.

*Gastrodia elata* Blume (GEB) is a valuable traditional Chinese medicine with significant effects on nervous system-related diseases, such as headache, epilepsy, and stroke. It is the tuber of the orchid and is widely distributed in China, Japan, South Korea, Nepal, India, Russia, and other places (Chen, 2023). In 2023, the National Health Commission of the People’s Republic of China and the State Administration for Market Regulation jointly issued a document to include GEB in the list of substances that traditionally serve as both food and traditional Chinese medicinal botanical drugs (Sun-waterhouse et al., 2024; Cong, 2024). Chronic toxicity studies have indicated that GEB has no significant mutagenic or toxic properties (Lu et al., 2020). Currently, more than 100 GEB compounds have been identified and are mainly classified as aromatic compounds, steroidal compounds, and organic acids based on the structure of their parent nuclei (Gong et al., 2024). Among these compounds, aromatic compounds such as 4-hydroxybenzyl alcohol (HBA), gastrodin (GAS), and Parishin compounds are the characteristic components of GEB with various pharmacological activities. Previous literature has indicated that HBA promotes a neuroprotective effect *via* anti-apoptosis (Yu et al., 2010). GAS can treat or improve epilepsy, Alzheimer’s disease, cerebral ischemia-reperfusion (I/R) injury, *etc* (Xiao et al., 2023). Parishins can protect the myocardium by inhibiting the phosphorylation of JNK1 (Wang et al., 2020). Although accumulating evidence suggests that multiple bioactive constituents in GEB may confer protective effects against cerebral ischemia-reperfusion injury, the precise pharmacodynamic substances responsible for its therapeutic efficacy remain to be elucidated. As a time-honored herbal medicine with predominant empirical applications, critical knowledge gaps persist regarding the specific bioactive entities that ultimately mediate its pharmacological actions *in vivo*, particularly their absorption patterns, metabolic transformations, and biodistribution dynamics within cerebral.

Pharmacokinetic characterization is pivotal for deciphering the therapeutic mechanisms of herbal medicines. The spatiotemporal distribution of bioactive compounds in target organs directly determines their pharmacological efficacy. Notably, disease-induced pathophysiological alterations—such as gastrointestinal dysmotility and BBB compromise in cerebral ischemia (Wang et al., 2024) - may substantially modify drug disposition parameters (Liu et al., 2022). This underscores the imperative to investigate disease-state pharmacokinetics for optimizing therapeutic regimens. Concurrently, the evolving advancements in high-resolution mass spectrometry have substantially augmented the capability for xenobiotic metabolite identification, particularly demonstrating unique advantages in tracing the biotransformation pathways of herbal medicine parent components (Wang et al., 2022).

**FIGURE 1:**
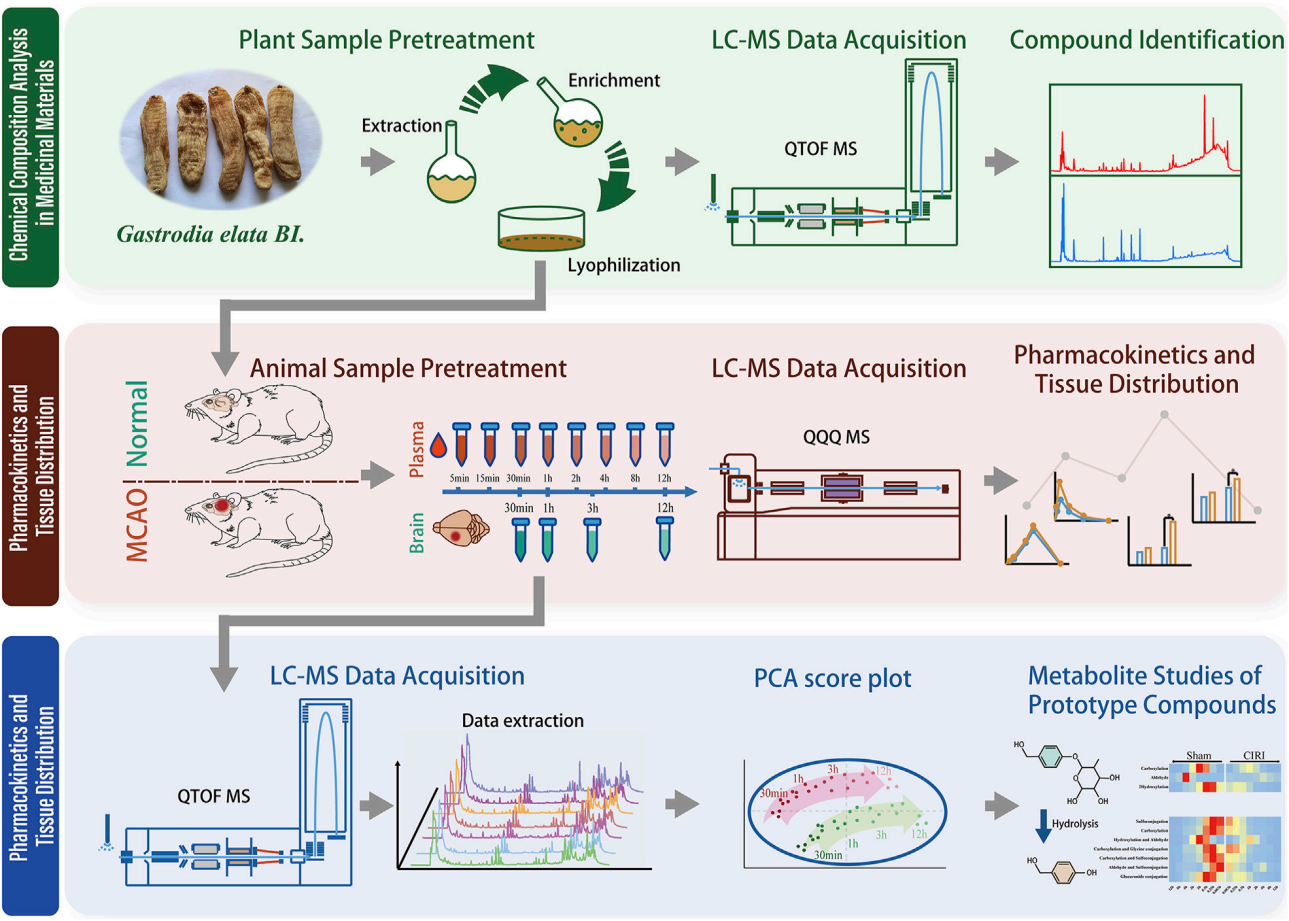
Graphical abstract.

Building upon preliminary research indicating GEB’s therapeutic potential for cerebral ischemia, we performed the following experiments: 1) Identification of GEB chemical constituents using UPLC-QTOF-MS; 2) Analysis of pharmacokinetic profiles and tissue distribution in both normal rats and rats with cerebral ischemia-reperfusion injury (CIRI) via UPLC-MS/MS; and 3) Identification of xenobiotic metabolites in rats using UPLC-QTOF-MS. The graphical abstract of the experimental workflow is shown in Figure 1. Through an integrated comparative analysis of component absorption kinetics, cerebral distribution characteristics, and exogenous metabolites, this study aims to elucidate the *in vivo* metabolic trajectory of GEB constituents, providing a pharmacokinetic foundation for subsequent pharmacodynamic research targeting CIRI.

## 2 Materials and METHODS

### 2.1 Reagents and chemicals

GEB tubers were procured from Ningbo Mingbei Chinese Pharmaceutical Co., Ltd. (Batch: 202,210; Origin: Shangluo, Shaanxi Province, China). The botanical material was authenticated as the dried tuber of *Gastrodia elata* Bl. through morphological and microscopic characterization by Mr. Guo Zengxi, Chief Pharmacologist of Traditional Chinese Medicine at Zhejiang Institute for Food and Drug Control. The crude drug (2 kg) was subjected to three successive reflux extractions with 75% aqueous ethanol (solvent-to-material ratio 10:1 v/w, 1 h per cycle), followed by combined filtration and concentration using rotary evaporation. The resultant extract was subsequently lyophilized, yielding 441.7 g of standardized extract (22.1% w/w yield). Quantitative analysis via ultra-performance liquid chromatography revealed the following constituent profile: Parishin A (PA, 6.11%), Parishin B (PB, 3.10%), Parishin C (PC, 0.57%), Parishin E (PE, 3.44%), gastrodin (GAS, 1.28%), S-(4-hydroxybenzyl) glutathione (4-HBG, 0.60%), and 4-hydroxybenzyl alcohol (HBA, 0.77%).

**FIGURE 2:**
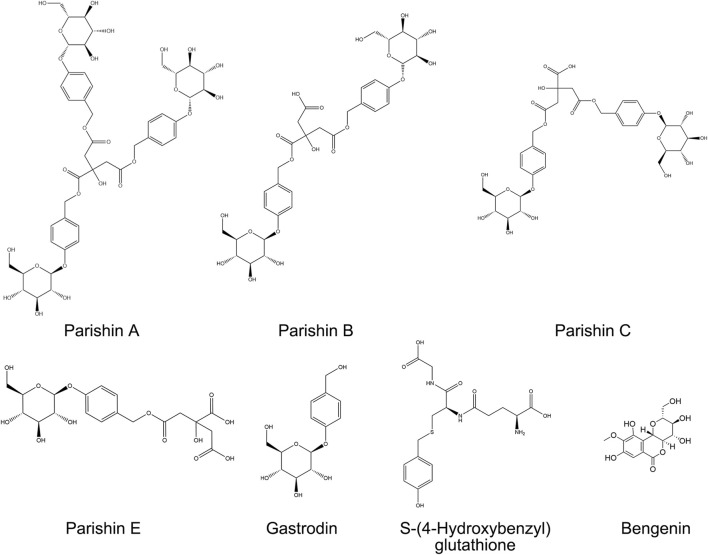
Chemical structures of PA, PB, PC, PE, GAS, 4-HBG, and Bergenin (IS).

Reference standards were obtained as follows: PA from Yuanye Bio-Technology Co., Ltd. (Shanghai, China; purity >98%); PB, PC, PE, and 4-HBG from Weikeqi Bio-Technology Co., Ltd. (Chengdu, China; purity >98%); GAS and bergenin (internal standard, IS) from the National Institute for Food and Drug Control (Beijing, China; purity >98%). HPLC-grade methanol, acetonitrile, and formic acid were purchased from Merck KGaA (Darmstadt, Germany). Deionized water was generated using a Milli-Q Integral Water Purification System (MilliporeSigma, United States). Chemical structures of analytes and IS are provided in Figure 2.

### 2.2 *In vivo* analyses

Male Sprague-Dawley rats (body weight 200–220 g) were procured from the Laboratory Animal Center of Hangzhou Medical College [Laboratory Animal Quality Certificate No. SCXK (Zhe) 2019–0,011]. Animals were housed in a specific pathogen-free (SPF) facility maintaining controlled environmental conditions: ambient temperature 22°C ± 2°C, relative humidity 50% ± 10%, and 12/12 h light/dark cycle with *ad libitum* access to standardized rodent chow and autoclaved water. Adaptive feeding was carried out for a week before the analyses, followed by a 12 h fast without prohibiting water. All animal-related protocols were carried out per the Laboratory animals care and use guidelines. Terminal anesthesia was achieved via intraperitoneal injection of pentobarbital sodium (40 mg/kg), followed by confirmation of euthanasia through cervical dislocation.

The experimental cohort was randomly allocated into two groups: Normal controls (n = 20) and middle cerebral artery occlusion (MCAO) models (n = 20). Cerebral ischemia was induced using Longa’s intraluminal filament technique under pentobarbital sodium anesthesia (40 mg/kg, i. p.). Briefly, a silicone-coated 4–0 nylon monofilament was advanced through the right external carotid artery to occlude the middle cerebral artery origin. After 120 min of ischemia, reperfusion was initiated by filament withdrawal. Neurological function was evaluated at 24 h post-reperfusion using the modified Neurological Severity Score (mNSS) system, which quantifies sensorimotor deficits through a 14-point composite scale assessing reflexes, balance, and motor coordination. Animals demonstrating moderate neurological impairment (mNSS 7–12) were included for subsequent interventions, while those with lethal scores (>12) or unsuccessful occlusion (mNSS <7) were excluded. Selected MCAO rats were administered standardized GEB extract (1,200 mg/kg, corresponding to 48 g crude drug per human daily equivalent) by oral gavage once daily for three consecutive days.

### 2.3 Chromatographic and mass spectrometric conditions

#### 2.3.1 Qualitative mass spectrometry analysis

The Agilent 1,290 series UPLC system equipped with an Agilent 6545 Q-TOF-MS (California, United States) was employed for UPLC-Q-TOF-MS analysis. Before each analysis, the instrument was calibrated *via* a tuning fluid Lu et al., 2019. Furthermore, system control, data acquisition, and processing were managed by MassHunter Workstation Software (Version B.08.00). Electrospray ionization was employed for recording mass spectra in negative and positive ion modes as well as with the auto MS/MS option. Moreover, Precursor and fragment ions with mass-to-charge ratios of 50–1,500 and 100–1,200 m/z mass-to-charge ratios were identified, respectively. The flow rate was 8 L/min, while the ion source gas temperature was 320°C. For the nebulizer, nitrogen was employed with 35 psi pressure. The respective capillary, skimmer, and fragmentor voltage were 3,500, 65, and 135 V. The collision energies were 20 and 40 eV.

The employed chromatographic column was a Waters CORTECS T3-C18 column (2.7 μm, 150 × 4.6 mm), with 35°C. Mobile phase A consisted of water containing 0.1% (v/v) formic acid, and mobile phase B was acetonitrile. For GEB as well as plasma and brain samples, different gradient elution procedures were set up for different analytical purposes.

The compound species and structure of GEB were comprehensively identified. Gradient elution was set as follows: 1% B for 0–10 min, 1%–10% B for 10–18 min, 10%–30% B for 18–35 min, 30%–60% B for 35–40 min, 60%–95% B for 40–60 min, 95%–1% B for 60–61 min, lastly, the gradient was restored to 1% B and then stopped at 70 min with a flow rate of 0.3 mL/min. The sample was injected at 1 μL volume. The data were acquired *via* positive and negative ion modes.

For the rapid identification of xenobiotic metabolites in plasma and brain tissue, gradient elution was performed as follows: 3%–35% B for 0–10 min, 35%–95% B for 10–20 min, 95%–3% B for 20–20.1 min, and then the gradient was restored to 3% B and stopped at 25 min with a flow rate of 0.5 mL/min. The negative ion collection mode was applied, and the sample injection volume was 4 μL.

#### 2.3.2 Quantitative mass spectrometry analysis

For this analysis, the Shimadzu 8,050 triple-quadrupole mass spectrometer and Shimadzu LC-30AD UPLC (Kyoto, Japan) equipped with an ESI interface were employed. Negative ion multiple reaction monitoring (MRM) mode was utilized for mass spectrometer settings. The instrument parameters were as follows: interface voltage = 3.0 kV, nebulizer gas flow = 3.0 L/min, heating gas flow = 10.0 L/min, drying gas flow = 10.0 L/min, DL temperature = 200°C, interface temperature = 300°C, heat block temperature = 400°C. The precursor-product ion pairs of the optimized MRM for the compounds and the internal standard are shown in Supplementary Table S1.

For the chromatography, a waters CORTECS T3-C18 column (2.7 μm; 150 × 4.6 mm) was selected, and the column temperature was set at 40°C. The mobile phase comprised (A) 0.02% formic acid water and (B) acetonitrile. The gradient elution was as follows: 3% B for 0–4.5 min, 3%–8% B for 4.5–5 min, 8%–30% B for 5–11 min, 30%–95% B for 11–12 min, 95%–3% B for 12–12.1 min, and back to 3% B for 16 min. The sample size was 4 μL, and the flow rate was 1 mL/min.

### 2.4 Pharmacokinetic sample processing

Eight behaviorally qualified rats from each group (Normal vs. MCAO, n = 8/group) received standardized GEB extract (1,200 mg/kg, equivalent to 48 g crude drug per human daily dose) via oral gavage once daily for three consecutive days. Serial blood sampling (0.5 mL) was performed via jugular vein cannulation at pre-dose (0 h) and 5 min, 15 min, 30 min, 1 h, 2 h, 4 h, 8 h, and 12 h post-final administration. Blood samples were immediately transferred to EDTA-K2 anticoagulant tubes, followed by centrifugation at 3,000 × g for 10 min at 4°C using a refrigerated centrifuge. The resultant plasma was aliquoted into polypropylene tubes.

### 2.5 Tissue distribution sample processing

An independent cohort (n = 6/group) was sacrificed under deep anesthesia (pentobarbital sodium 40 mg/kg, i. p.) at 0.5 h, 1 h, 3 h, and 12 h post-final dosing. Cerebral perfusion was performed with ice-cold normal saline via left ventricular cannulation to eliminate residual blood. Whole brains were rapidly excised, weighed, and homogenized in four volumes of phosphate-buffered saline (pH 7.4) using a tissue grinder (Precellys 24, Bertin Technologies, France). Homogenates were centrifuged at 12,000 × g for 15 min at 4°C. The supernatants were filtered through 0.22 μm PVDF membranes and aliquoted into polypropylene tubes.

### 2.6 Preparation of animal plasma and brain tissue samples

The literature has indicated that the protein precipitation method has a higher recovery rate than the liquid-liquid extraction protocol and is easier to operate than the solid-phase extraction technique (Liu et al., 2023).

Take 200 µL of rat plasma, add 100 µL of internal standard solution and 300 µL of ice-cold methanol, then vortex-mix for 5 min. Centrifuge the mixture at 12,000 rpm for 10 min at 4°C. Collect the supernatant and filter it through a 0.22 µm microporous membrane to obtain the final sample. For UPLC-MS/MS analysis, 4 μL of the supernatant was injected.

Weigh 300 mg of rat brain tissue and add 3 volumes of PBS solution. Homogenize the mixture at 70 Hz for 90 s, followed by centrifugation at 12,000 rpm for 10 min at 4°C. Collect the supernatant to obtain the test tissue homogenate. Take 400 µL of the homogenate, add 50 µL of internal standard solution and 1,150 µL of methanol, then vortex-mix for 5 min. Centrifuge the mixture at 12,000 rpm for 10 min at 4°C. Collect 1,200 µL of the supernatant and dry it under a nitrogen stream. Reconstitute the residue in 300 µL of methanol, centrifuge again, collect the supernatant, and filter through a 0.22 µm microporous membrane to obtain the final sample. For UPLC-MS/MS analysis, 4 μL of the supernatant was injected.

### 2.7 Preparation of stock and working solutions

#### 2.7.1 Standard stock solutions

The stock solution was prepared by dissolving 6 precisely weighed reference materials in methanol. The stock solution was then mixed and diluted with methanol to acquire a series of standard working solutions. Bergenin (120 ng/mL) was dissolved in methanol and configured as an internal standard (IS).

#### 2.7.2 Plasma calibration standards & QC samples

Calibration curves were constructed by spiking 20 μL of analyte working solution into 180 μL drug-free rat plasma, yielding six concentration levels. Quality control samples (QCs) at low (L), medium (M) and high (H) levels were prepared in the same manner. Spiked samples were vortex-mixed (1,500 rpm, 1 min) and processed identically to experimental samples through protein precipitation with ice-cold methanol (1:3 v/v).

#### 2.7.3 Brain tissue calibration standards & QC samples

For cerebral quantification, 40 μL of the composite working solution was added to 360 μL blank brain homogenate, establishing six-point calibration curves. Quality controls (QCs) at three levels (L, M, H) were prepared similarly. Homogenate-spiked samples underwent identical homogenization and centrifugation protocols as described in Section 2.6.

The specific concentrations of the prepared calibration standards and QC samples are provided in Supplementary Tables S2–S3.

### 2.8 Method validation

Based on the ICH M10 “Bioanalytical Method Validation”, the method was validated to determine 6 components in the biological matrix, including specificity, recovery, linearity, accuracy, precision, lower quantification limit, stability, and matrix effect.

#### 2.8.1 Specificity

The chromatograms of blank matrix samples from six rats, blank biosamples supplemented with control and IS, and biosamples of rats treated with GEB extract were analyzed for specificity analysis to minimize potential interference from endogenous substances.

According to ICH M10 guidelines, the analyte and IS retention sites of blank matrix samples should not show endogenous reactions. For each matrix, the interfering substance response should not exceed 5% of the IS response or 20% of the analyte response in the lower limit of quantification (LLOQ) sample.

#### 2.8.2 Linearity and lower quantitation limits

The calibration curves of the peak area ratio of the pooled analyte and IS against each analyte’s concentration were plotted *via* Least-squares linear regression (weighted factor: 1/x^2^). When the regression coefficient r of the calibration curve was ≥ 0.99, the requirement was met. The LLOQ was described as the lowest concentration in the calibration curve that could be quantitatively measured when the signal-to-noise ratio was > 10.

#### 2.8.3 Accuracy and precision

The accuracy and intra-day precision were investigated by repeated measurements of QC samples at 3 concentrations (L, M, H) daily, analyzed in six replicates per day over three consecutive days. Furthermore, accuracy and precision were depicted by relative error (RE) and relative standard deviation (RSD), respectively. The RSD of QC should be < 15% and RE within ± 15%.

#### 2.8.4 Recovery and matrix effect

At the 3 concentration levels (L, M, H), the recovery and matrix effects of the components were determined using six replicates of QC samples. Recovery was determined by comparing the peak areas of before extraction samples to post-extracted spiked samples at three QC concentrations. The matrix effect is the peak response ratio between the component dissolved in the mobile phase at various concentrations and the component dissolved in the blank biological matrix. Acceptable RSD for recovery and matrix effects was set as ≤ 15%.

#### 2.8.5 Stability

Stability was elucidated by assessing the QC sample’s (L, M, H) stability in different storage and handling conditions, such as at - 80°C for 1 month (long-term) storage and 3 freeze-thaw cycles. The RSD of QC should be < 15% and RE within ± 15%.

### 2.9 Drug configuration

High-dose GEB extract was prepared by adding 4.8 g of GEB extract in 40 mL of purified water to make a 120 mg/mL solution (calculated based on the human daily intake of 48 g of GEB).

The low dose of GEB was prepared by adding 2 g of GEB extract in 40 mL of purified water to make a 50 mg/mL solution (calculated based on the human daily intake of 20 g of GEB).

For preparing positive drugs, 2 nimodipine tablets (30 mg/tablet) were ground and added to 50 mL of purified water to make a 1.2 mg/mL solution (calculated based on the human daily intake of 120 mg of nimodipine).

All the solutions were stored at −40°C for no more than 1 week.

### 2.10 TTC staining

After induction of deep anesthesia with pentobarbital (40 mg/kg), the rats were euthanized by cervical dislocation, followed by decapitation for brain removal. The brains were immediately flash-frozen in a freezer for 20 min. Then, the olfactory bulb and cerebellum were removed, the brain was cut into 6 coronal sections from anterior to posterior, stained with 1% TTC solution in the dark for 20 min at 37°C, and preserved in 4% paraformaldehyde for 30 min.

### 2.11 Xenobiotic metabolite studies

The metabolism of all identified compounds was studied based on the simple decomposition pathway of the main compounds in GEB: PA→ PB/PC→ PE/PG→ GAS → 4-HBA.

The xenobiotic metabolite investigation protocol comprised three sequential phases: (1) Raw MS data stratification by experimental groups (Normal/MCAO) and temporal sampling points (0–12 h) was first performed to establish cohort-specific metabolic trajectories; (2) An algorithmic workflow in MassHunter Profinder (vB.10.0) then executed feature extraction through peak detection (>300 counts), retention time alignment (ΔRT = 0.05% + 0.15 min), mass calibration (Δm/z = 20 ppm + 2.0 mDa), and adduct annotation ([M-H]^-^/[M + HCOO]^-^), exporting processed data as. csv files; (3) A xenobiotic metabolite prediction database was constructed using R (v4.1.1) to screen candidate components with a mass tolerance of ±10 ppm, followed by structural confirmation through fragmentation pattern analysis, MS/MS spectral matching, and retention time consistency validation. The R code and detailed MS/MS structural confirmation process for xenobiotic metabolites are provided in Supplementary Data.

### 2.12 Data analysis

LabSolutions LCMS Version 5.118 (Kyoto, Japan) was utilized to quantify 6 parent compounds. Pharmacokinetic parameters, time to reach the maximum concentration (T_max_), clearance rectified by bioavailability (CL_z/F_), mean resident time (MRT), including area under the plasma concentration-time curve (AUC), elimination half-life (T_1/2_), apparent volume of distribution rectified by bioavailability (V_z/F_) were analyzed by a non-compartmental method using Winnonlin 8.10 software (Pharsight Co., United States) software. Pharmacokinetic parameters were depicted as mean ± standard deviation (SD) (x ± s). All the statistical analyses were carried out *via* GraphPad Prism Version 10.0 software, and *p* < 0.05 was deemed statistically significant.

## 3 Results

### 3.1 Evaluation of the therapeutic effect of GEB on cerebral ischemia in rats

**FIGURE 3:**
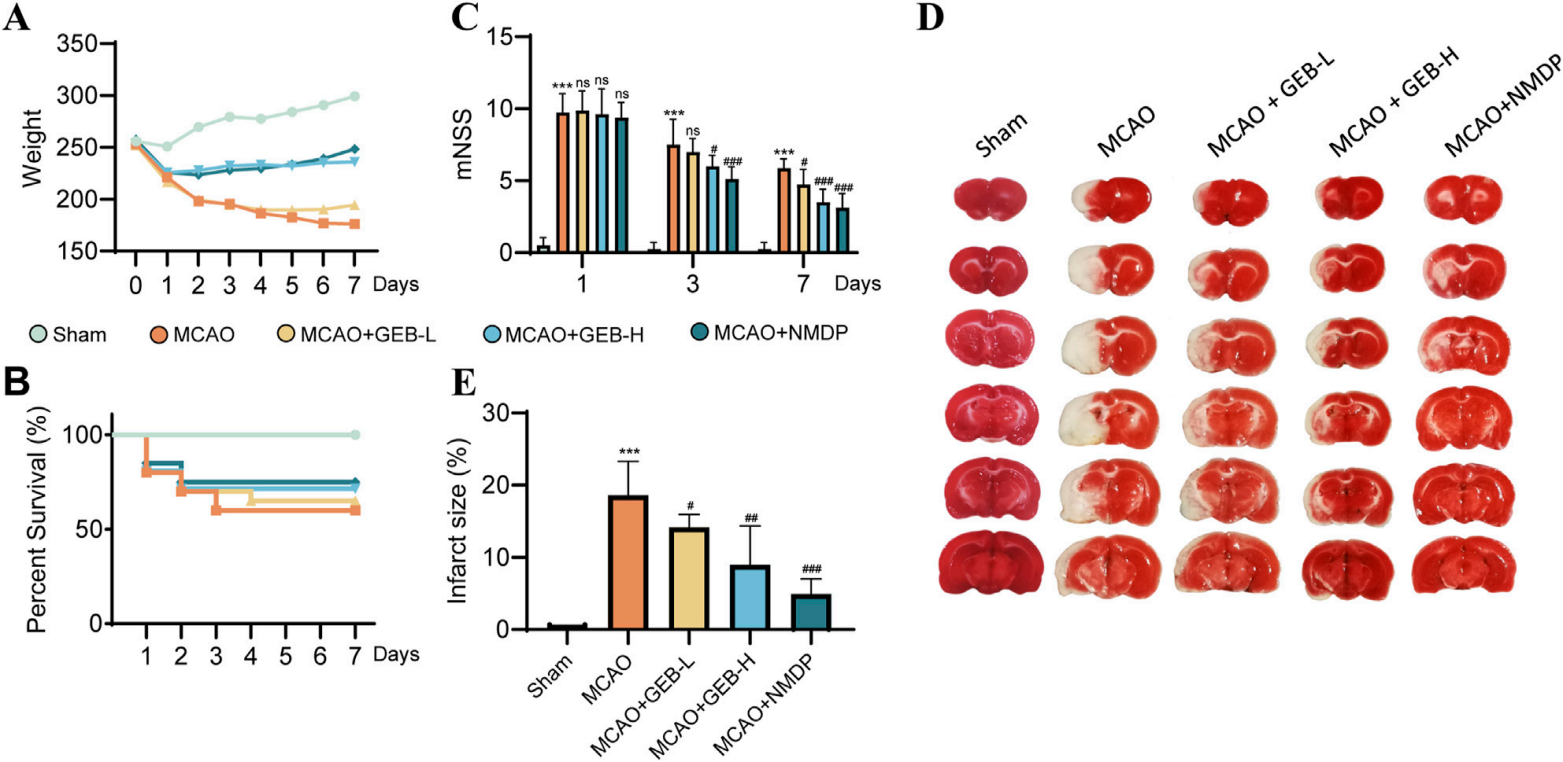
Evaluation of the therapeutic effect of GEB extract on cerebral ischemia in rats (mean ± SEM, n = 6). Azure represents Sham group, orange represents MCAO group, yellow represents GEB-L group, skyBlue represents GEB -H group, darkBlue represents NMDP group. ^***^ *P* < 0.005 vs Sham group. ^*#*^ *P* < 0.05, ^*##*^ *P* < 0.01, ^*###*^ *P* < 0.005 vs MCAO group. **(A)** Weight monitoring for 1 week. **(B)** Survival curves within a week. **(C)** mNSS score. **(D,E)** TTC staining.

Rats were allocated into five groups: sham-operated (Sham), MCAO, low-dose GEB extract (GEB-L), high-dose GEB extract (GEB-H), and nimodipine control (NMDP). Model validity was confirmed via modified Neurological Severity Score (mNSS), with rats demonstrating moderate neurological impairment (scores 7–12) on post-operative day 1 selected for subsequent experiments. Longitudinal monitoring included body weight tracking and survival assessment, complemented by triphenyltetrazolium chloride (TTC) staining for cerebral infarction evaluation at study termination. The MCAO group exhibited characteristic ischemic pathology: progressive neurological deterioration, significant weight loss, and extensive infarction; in contrast, both GEB and NMDP improved these outcomes, with the GEB-H showing significantly superior efficacy to the GEB-L group, indicating its dose-dependent therapeutic effects (Figures 3A–E).

As evidenced by previous studies (Wang et al., 2022), the 1–3 day period following cerebral ischemia constitutes the acute phase, characterized by severe cerebral edema, pro-inflammatory cytokine storm, and progressive BBB disruption. This transitions into the subacute phase (days 3–7), marked by a homeostatic balance between pro- and anti-inflammatory signaling that initiates repair mechanisms, with day 3 post-ischemia representing a critical transition point in BBB breakdown/recovery dynamics Shi et al., 2012. Based on previous findings, we also observed that, except for the sham group, deaths occurred in varying degrees among the other groups of rats within 1–3 days after surgery. After day 3, mortality was observed in only one group. The mNSS results indicated that the model group maintained scores above 7 on postoperative day 3, reflecting severe brain injury in these rats. By day 7, all groups showed improvement in behavioral scores with time. Given this biphasic pathology, our investigation employed a 3-day high-dose GEB extract regimen to systematically compare the pharmacokinetic profiles and cerebral biodistribution patterns between normal and MCAO model rats Lu et al., 2019.

### 3.2 Identification of chemical compounds in GEB extracts

**FIGURE 4:**
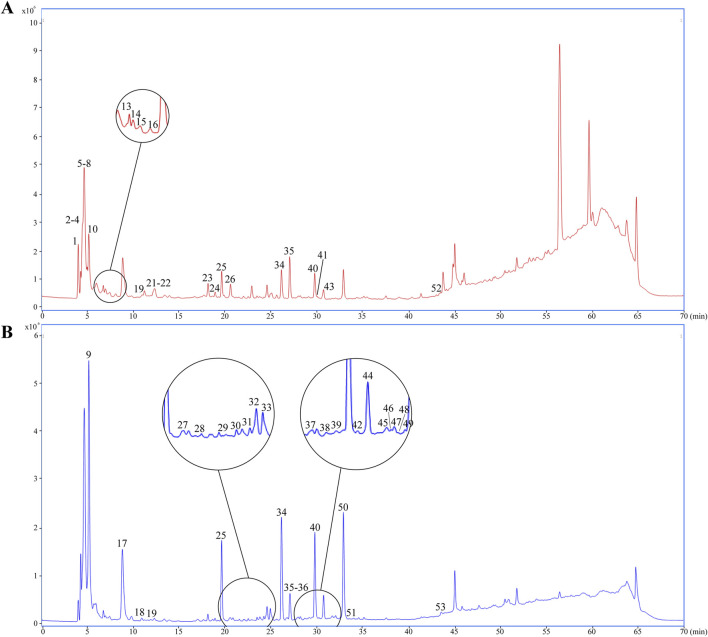
Dentification of chemical composition in GEB extract by UPLC-Q-TOF-MS. **(A)** Plot of the TIC in positive ion mode. **(B)** Plot of the TIC in negative ion mode.

UPLC-Q-TOF-MS analysis was systematically performed in dual ionization modes (positive/negative) to elucidate the phytochemical profile of GEB extracts. As shown in the total ion chromatograms (TICs, Figure 4), 53 characteristic constituents were identified through accurate mass measurement (<10 ppm error), diagnostic fragment ions, and chromatographic retention behavior, including 16 amino acids, 14 parishin-type compounds, GAS, and HBA. Mechanistic studies have revealed that signature phenolic constituents in GEB - particularly HBA, GAS, and parishin derivatives - demonstrate multifunctional bioactivities encompassing mitochondrial apoptosis inhibition, antidepressant effects, and marked anti-inflammatory efficacy, constituting the principal pharmacologically active components underlying GEB’s therapeutic actions (Jiang et al., 2014; Zhang et al., 2012). The complete phytochemical inventory with structural annotations is provided in Table 1.

**TABLE 1:** Details of GEB extract compounds.

NO.	Compound	Formula	RT (min)	Mode	Theoretical mass (Da)	Measured (m/z)	Error (ppm)	Ion fragment	References
1	*L-*Arginine	C6H14N4O2	4.37	[M + H]^+^	174.1117	175.1185	−5.72	158.0920、130.0963、116.0701、70.0654	Fan et al. (2022)
2	*L*-Serine	C3H7NO3	4.42	[M + H]^+^	105.0426	106.0497	−6.78	88.0387、70.0285、62.0541、60.488	Kim et al. (2023)
3	Asparagine	C4H8N2O3	4.44	[M + H]^+^	132.0535	133.0605	−6.15	116.0338、88.0391、74.0237	Kim et al. (2023)
4	Aspartic acid	C4H7NO4	4.48	[M + H]^+^	133.0375	134.0448	−3.98	88.0393、70.0290	Kim et al. (2023)
5	*L*-Threonine	C4H9NO3	4.53	[M + H]^+^	119.0583	120.0651	−8.07	102.0566、84.0457、74.0599	Kim et al. (2023)
6	*L-*Valinol	C5H13NO	4.55	[M + H]^+^	103.0997	104.1068	−7.1	58.0647	Fan et al. (2022)
7	Glutamic acid	C5H9NO4	4.64	[M + H]^+^	147.0532	148.0609	−0.57	102.0545、84.0445、56.0499	Kim et al. (2023)
8	*L-*Proline	C5H9NO2	4.99	[M + H]^+^	115.0634	116.0703	−7.36	70.0649、58.0648	Fan et al. (2022)
9	Sucrose	C12H22O11	5.15	[M-H]^-^	342.1162	341.1083	−0.26	179.0573、89.0247	Li (2004)
10	Ergothioneine	C9H15N3O2S	5.16	[M + H]^+^	229.0885	230.0953	−4.45	186.1057、127.0322、100.0215、60.0808	Kim et al. (2023)
11	Malic acid	C4H6O5	5.68	[M-H]^-^	134.0215	133.0147	7.52	115.0049、71.0146	Fan et al. (2022)
12	Valine	C5H11NO2	6.00	[M + H]^+^	117.0790	118.0860	−6.81	72.0809、58、0,650、55.0546	Kim et al. (2023)
13	Tropine	C8H15NO	6.71	[M + H]^+^	141.1154	142.1221	−7.66	124.1118	Kim et al. (2023)
14	Vitamin B4	C5H5N5	6.86	[M + H]^+^	135.0545	136.0618	−3.82	119.0344、92.0240、65.0141	Kim et al. (2023)
15	Methionine	C5H11NO2S	7.47	[M + H]^+^	149.0511	150.0578	−7.17	104.0524、87.0252、74.0231	Kim et al. (2023)
16	Nicotinamide	C6H6N2O	8.05	[M + H]^+^	122.0480	123.0551	−6	106.0283、96.0433、80.0491	Kim et al. (2023)
17	Pyroglutamic acid	C5H7NO3	9.00	[M-H]^-^	129.0426	128.0358	8.05	82.029	Kim et al. (2023)
18	Succinic Acid	C4H6O4	10.88	[M-H]^-^	118.0266	117.0198	8.67	99.9257、73.0304	Fan et al. (2022)
19	*L-*Leucine	C6H13NO2	11.19	[M + H]^+^	131.0947	132.1016	−6.46	86.0963、69.0700	Fan et al. (2022)
20	gastrodibezen B	C14H12O4	11.90	[M-H]^-^	244.0735	243.0638	−7.96	192.8602、163.7050、124.0387、110.0236	Wang et al. (2021)
21	*L-*Isoleucine	C6H13NO2	12.17	[M + H]^+^	131.0947	132.1016	−6.46	86.0963、69.0700	Fan et al. (2022)
22	Tyrosine	C9H11NO3	12.29	[M + H]^+^	181.0739	182.0807	−5.6	136.0752、123.0439、119.0488、91.0541	Kim et al. (2023)
23	Adenosine	C10H13N5O4	18.18	[M + H]^+^	267.0968	268.1038	−2.91	136.0613、119.0349	Kim et al. (2023)
24	Guanosine	C10H13N5O5	18.90	[M-H]^-^	283.0916	282.0860	7.64	150.0412、133.0512、108.0193	Kim et al. (2023)
25*	Gastrodin	C13H18O7	19.63	[M + COOH]^-^	286.1053	331.1059	9.03	123.0455、105.0346	Li et al. (2015)
25*	Gastrodin	C13H18O7	19.63	[M + NH_4_]^+^	286.1053	304.1395	−1.39	107.0492	Li et al. (2015)
26	*L-*Phenylalanine	C9H11NO2	20.61	[M + H]^+^	165.0790	166.0860	−4.84	120.0804、103.0539、77.0383	Wang et al. (2021)
27	Dactylose A	C12H16O6	20.85	[M-H]^-^	256.0947	255.0864	−1.82	165.0571、135.0430	Kizu et al. (1999)
28	Gastrodin A	C19H28O12	21.59	[M-H]^-^	448.1581	447.1503	0.1	341.1085、179.0546、123.0444、105.0340	Delaquis et al. (2005)
29*	HBA	C7H8O2	22.89	[M-H]^-^	124.0524	123.0455	7.27	77.0394	Tang et al. (2016)
30	Benzyl beta-D-glucopyranoside	C13H18O7	23.46	[M + COOH]^-^	286.1053	331.1049	6.01	123.0461、105.0349、101.0246	Ishikawa et al. (2002)
31	Glucosyringic acid	C15H20O10	24.27	[M-H]^-^	360.1056	359.0997	5.22	197.0543	Huang et al. (2006)
32	6 ′-p-hydroxyphenyl methanol ether gastrodia disaccharide	C19H28O12	24.35	[M-H]^-^	448.1581	447.1502	−0.12	341.1091、179.0569、161.0437	Li (2004)
33	Parishin G	C19H24O13	24.94	[M-H]^-^	460.1217	459.1131	−1.68	369.1339、113.0242、101.0238、71.0133	Li et al. (2015)
34*	Parishin E	C19H24O13	26.15	[M-H]^-^	460.1217	459.1131	−1.68	193.0260、173.0095、159.0277、111.0088	Li et al. (2015)
34*	Parishin E	C19H24O13	26.15	[M + NH_4_]^+^	460.1217	478.1561	−1.22	175.0228、107.0490	Li et al. (2015)
35*	S-(4-hydroxybenzyl) glutathione	C17H23N3O7S	27.04	[M-H]^-^	413.1256	412.1182	0.85	306.0761、272.0882、179.0454、111.0080	Andersson et al. (1995)
35*	S-(4-hydroxybenzyl) glutathione	C17H23N3O7S	27.04	[M + H]^+^	413.1256	414.1340	3.51	308.0913、179.0485	Andersson et al. (1995)
36	N-(4′-hydroxybenzyl)pyroglutamate	C12H13O4N	27.19	[M-H]^-^	235.0844	234.0763	−1.43	146.0241、128.0361	Guo et al. (2015)
37	Parishin I	C38H50O24	28.03	[M-H]^-^	890.2692	889.2655	4.63	727.2103、501.1306、423.0946、161.0497	Li et al. (2015)
38	Parishin J	C20H26O13	28.63	[M-H]^-^	474.1373	473.1287	−1.73	413.1083、143.0349、123.0458、111.0086	Li et al. (2015)
39	Parishin V	C38H50O24	29.54	[M-H]^-^	890.2692	889.2651	4.18	585.1642、217.0517、161.0471、111.0088	Li et al. (2015)
40	Parishin B	C32H40O19	29.78	[M-H]^-^	728.2164	727.2087	0.19	459.1158、423.0962、161.0465、111.0091	Huang et al. (2006)
40*	Parishin B	C32H40O19	29.78	[M + NH_4_]^+^	728.2164	746.2513	−1.47	299.0751、107.0490	Huang et al. (2006)
41	N6-(4-hydroxybenzyl)adenosine	C17H19O5N5	30.00	[M + H]^+^	373.1386	374.1460	−1.19	242.1032、136.0611、107.0488	Huang et al. (2006)
42	Parishin H/M	C33H42O20	30.23	[M-H]^-^	758.2269	757.2201	1.29	453.1080、161.0460.111.0084	Li et al. (2015)
43	Syringate	C9H10O5	30.60	[M + H]^+^	198.0529	199.0600	−3.27	140.0664、125.0375	Fang et al. (2023)
44*	Parishin C	C32H40O19	30.76	[M-H]^-^	728.2164	727.2087	0.19	459.1147、369.1223、161.0466、111.0089	Li et al. (2015)
45	4-Hydroxybenzaldehyde	C7H6O2	31.65	[M-H]^-^	122.0368	121.0298	6.98	92.0264	Tang et al. (2016)
46	Parishin N/O	C21H28O13	31.70	[M-H]^-^	488.1530	487.1452	0.06	183.0307、111.0097	Li et al. (2015)
47	*p*-Ethoxymethyl-phenyl-O-*β*-D-glucoside	C15H22O7	31.96	[M + COOH]^-^	314.1366	359.1335	−1.98	106.0638	Huang et al. (2006)
48	Parishin K	C33H42O19	32.07	[M-H]^-^	742.2320	741.2272	4.03	473.1306、423.0920、285.09868、123.0447	Li et al. (2015)
49	Parishin F	C51H66O30	32.61	[M-H]^-^	1,158.3639	1,157.3573	1.06	1,157.3573	Li et al. (2015)
50*	Parishin A	C45H56O25	32.90	[M-H]^-^	996.3111	995.3015	−1.76	727.2102、459.1137、369.1199、179.0555	Li et al. (2015)
51	Parishin L	C46H58O26	33.21	[M-H]^-^	1,026.3216	1,025.3156	1.74	727.2089、423.0936、161.0461	Li et al. (2015)
52	Cinnamic acid	C9H8O2	43.04	[M + H]^+^	148.0525	149.0600	−1.71	131.0478、103.0539	Kim et al. (2023)
53	Grossamide	C36H36N2O8	43.88	[M-H]^-^	624.2471	623.2386	−1.19	460.1738、297.1133、282.0888、146.9653	Huang et al. (2006)

Note: Mark * refers to compounds identified by reference substance.

### 3.3 Identification of components in rat plasma and brain tissue

**FIGURE 5:**
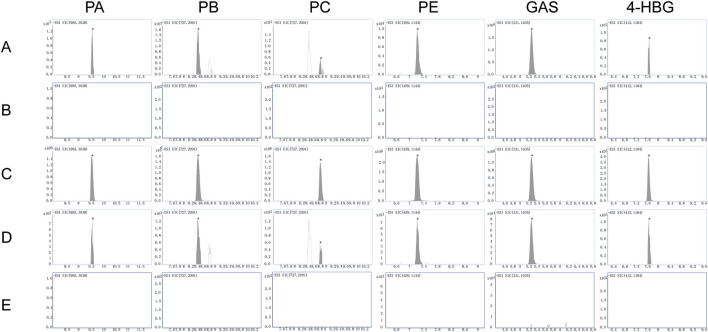
EICs plots in negative ion mode: **(A,B)** Plasma from rats treated with GEB extract versus vehicle-treated control rats. **(C)** Standard reference materials for various compounds. **(D,E)** Brain tissue from rats treated with GEB extract versus vehicle-treated control rats.

UPLC-Q-TOF-MS analysis in negative ion mode was systematically employed to detect prototypical components of GEB in plasma and cerebral tissues of dosed versus control rats. Six intact phytochemicals—PA, PB, PC, PE, GAS, and 4-HBG—were unequivocally identified through characteristic adducts, with chromatographic and spectral consistency against reference standards. As illustrated by the extracted ion chromatograms (EICs) plots in Figure 5, all identifications were validated via retention time alignment and MS/MS spectral matching, forming the foundation for subsequent pharmacokinetic and cerebral biodistribution studies.

### 3.4 Method validation

For the methodological validation, the sensitivity, linearity, specificity, matrix effect, precision, accuracy, and stability of the method were comprehensively assessed. The data revealed that the method was reliable, stable, and suitable for pharmacokinetics and brain tissue distribution analysis.

#### 3.4.1 Specificity

**FIGURE 6:**
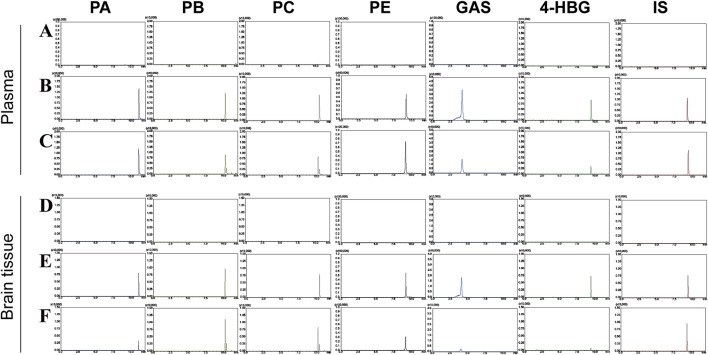
MRM chromatograms of PA, PB, PC, PE, GAS, 4-HBG, and IS under negative ion conditions. **(A)** blank matrix plasma; **(B)** blank matrix plasma spiked with mixed reference materials; **(C)** rat plasma 1h after GEB extract administration; **(D)** blank matrix brain tissue; **(E)** brain tissue spiked with the mixed reference material; **(F)** brain tissue of rats at 1 h after administration of GEB extract.

The MRM chromatograms of blank matrix plasma spiked with standard mixed standard material, blank matrix plasma, rat plasma, blank matrix brain tissue, and brain tissue spiked with standard mixed standard material 1 h after administrating GEB extract are shown in Figure 6. No interference peaks were observed from endogenous components near the retention time for the six selected compounds and IS. These data showed that the analyte had good specificity in rat plasma and brain tissue.

#### 3.4.2 Linearity and lower limits of quantitation (LLOQ)

Equations for correlation coefficients, calibration curves, and lower limits of quantification for PA, PB, PC, E, GAS, and 4-HBG in brain tissue and plasma are shown in Table 2. Furthermore, excellent linearity was observed over the concentration range of all biological samples with a correlation coefficient (*r* ^2^) > 0.99.

**TABLE 2:** The regression equation, linear range, *r* ^2^, and LLOQ of six analytes in plasma and brain tissue (n = 6).

Sample	Compounds	Regression equations	Linear range (ng)	LLOQ (ng/mL)	LOD (ng/mL)	*r* ^2^
Plasma	PA	Y = 0.0307X + 0.0126	1.86–236.76	1.86	0.60	0.9996
	PB	Y = 0.0243X - 0.0040	1.89–240.75	1.89	0.60	0.9996
	PC	Y = 0.0294X + 0.0130	1.80–230.76	1.80	0.60	0.999
	PE	Y = 0.0451X + 0.0050	1.17–741.75	1.17	0.45	0.9999
	GAS	Y = 0.0039X + 0.1186	21.51–13,775.01	21.51	7.40	0.9996
	4-HBG	Y = 0.0733X - 0.0378	1.59–204.51	1.59	0.60	0.9995
Brain tissue	PA	Y = 0.0310X - 0.0007	0.62–78.92	0.62	0.20	0.9997
	PB	Y = 0.0267X - 0.0139	0.63–80.25	0.63	0.20	0.9998
	PC	Y = 0.0223X - 0.0159	0.6–76.92	0.60	0.20	0.9994
	PE	Y = 0.0716X - 0.0634	0.39–247.25	0.39	0.15	0.9997
	GAS	Y = 0.0048X + 0.0677	7.17–4,591.67	7.17	2.80	0.9998
	4-HBG	Y = 0.0817X - 0.0753	0.53–68.17	0.53	0.20	0.9993

#### 3.4.3 Accuracy and precision

The intra- and inter-day precision of plasma samples was 93.25%–110.32% and 85.24%–109.81%, respectively, while that of the brain tissue samples was 91.99%–108.94% and 85.56%–107.09%, respectively (Table 3). The RE and RSD were within the acceptable standard range, indicating that the brain tissue and plasma samples had good stability and the analytical method had good repeatability.

**TABLE 3:** Intra-day and inter-day precision of PA, PB, PC, PE, GAS, and 4-HBG in plasma and brain tissue (n = 6).

Compounds	Plasma							Brain tissue						
	Concentration (ng/mL)	Intra-day precision			Inter-day precision			Concentration (ng/mL)	Intra-day precision			Inter-day precision		
		Accuracy (%)	SD (%)	RSD (%)	Accuracy (%)	SD (%)	RSD (%)		Accuracy (%)	SD (%)	RSD (%)	Accuracy (%)	SD (%)	RSD (%)
PA	236.75	93.25	6.77	7.26	85.24	5.54	6.50	78.92	99.63	7.76	7.79	90.63	4.50	4.97
	59.19	93.29	6.64	7.12	85.86	7.55	8.79	19.73	103.62	11.24	10.85	94.54	8.53	9.03
	7.40	101.1	6.90	6.83	90.39	9.84	10.88	2.47	94.79	14.43	15.23	101.60	13.48	13.26
PB	240.75	97.80	5.30	5.42	92.91	4.82	5.19	80.25	97.70	5.11	5.23	90.65	3.17	3.49
	60.19	102.26	7.76	7.59	102.33	6.69	6.53	20.06	97.50	4.41	4.52	92.06	7.03	7.64
	7.52	101.30	8.55	8.44	96.35	7.77	8.07	2.51	104.04	9.94	9.56	100.16	6.83	6.82
PC	230.75	99.17	5.86	5.91	105.76	6.94	6.56	76.92	97.45	2.71	2.78	92.68	6.17	6.66
	57.69	97.93	6.36	6.50	101.99	7.20	7.06	19.23	97.40	9.10	9.35	85.56	6.41	7.50
	7.21	91.11	7.57	8.31	94.23	7.61	8.07	2.40	101.85	11.54	11.33	95.85	11.89	12.41
PE	741.75	107.97	4.96	4.59	109.81	5.59	5.09	247.25	99.99	6.57	6.57	93.82	5.44	5.79
	185.44	107.38	5.44	5.07	107.10	4.33	4.04	61.81	101.84	5.07	4.97	96.49	6.56	6.79
	11.59	94.46	6.88	7.28	98.25	3.54	3.60	3.86	94.40	2.42	2.56	92.67	8.60	9.28
GAS	13,775.00	99.57	4.88	4.90	102.48	3.91	3.81	4,591.67	104.88	4.97	4.74	107.09	4.94	4.61
	3,443.75	105.89	3.00	2.84	102.67	3.91	3.80	1,147.92	107.99	6.46	5.98	105.28	4.26	4.05
	215.23	99.83	7.42	7.43	92.50	3.05	3.29	71.74	108.94	4.87	4.47	106.89	7.75	7.25
4-HBG	204.50	104.35	4.14	3.96	105.01	5.79	5.52	68.17	98.51	4.67	4.74	91.66	5.87	6.4
	51.13	110.32	5.67	5.14	106.23	5.17	4.87	17.04	98.18	9.48	9.66	90.58	5.18	5.72
	6.39	96.10	7.27	7.57	100.67	6.28	6.24	2.13	91.99	12.65	13.76	87.78	5.02	5.72

#### 3.4.4 Recovery and matrix effect

The matrix effect of all plasma samples ranged between 86.26% and 114.26%, while that of the brain samples ranged from 91.58% to 112.50%. Furthermore, their RE and RSD were within the acceptable standard range. Moreover, the endogenous substances in both plasma and brain tissue samples did not significantly affect the ionization efficiency of the analyte (Table 4).

**TABLE 4:** Matrix effects of PA, PB, PC, PE, GAS, and 4-HBG in Plasma and Brain tissue (n = 6).

Compounds	Plasma				Brain tissue			
	Concentration (ng/mL)	Matrix effect (%)	SD (%)	RSD (%)	Concentration (ng/mL)	Matrix effect (%)	SD (%)	RSD (%)
PA	236.76	90.69	4.50	4.97	78.92	93.72	4.87	5.19
	59.19	93.04	1.77	1.91	19.73	97.39	8.76	9.00
	7.41	92.06	6.93	7.53	2.47	106.62	11.39	10.68
PB	240.75	106.25	2.05	1.93	80.25	100.17	7.76	7.75
	60.18	105.14	2.68	2.55	20.06	109.07	5.67	5.20
	7.53	108.24	12.25	11.32	2.51	106.54	12.99	12.19
PC	230.76	109.06	3.66	3.35	76.92	105.93	3.16	2.98
	57.69	108.34	8.54	7.88	19.23	107.93	8.54	7.91
	7.20	114.26	11.96	10.47	2.40	93.28	8.54	9.15
PE	741.75	106.28	4.93	4.63	247.25	101.07	7.40	7.32
	185.43	106.25	3.46	3.26	61.81	106.71	5.78	5.42
	23.19	104.86	6.46	6.16	3.86	98.20	6.27	6.39
GAS	13,775.01	99.57	3.12	3.13	4,591.67	91.58	6.56	7.16
	3,443.76	93.65	1.81	1.93	1,147.92	97.05	5.56	5.73
	215.25	86.26	4.78	5.54	71.74	93.53	2.23	2.39
4-HBG	204.51	106.28	2.94	2.77	68.17	102.04	8.17	8.01
	51.12	108.17	2.04	1.88	17.04	112.50	4.65	4.13
	6.39	111.18	10.37	9.33	2.13	106.95	10.39	9.72

The recovery of all plasma samples ranged between 96.23% and 108.77%, whereas that of the brain tissue sample was between 96.57% and 106.51%. Their RE and RSD were within the acceptable standard range, suggesting that plasma and brain tissue analysis results had a certain reliability (Table 5).

**TABLE 5:** Recovery of PA, PB, PC, PE, GAS, and 4-HBG in plasma and brain tissue (n = 6).

Compounds	Plasma				Brain tissue			
	Concentration (ng/mL)	Recovery (%)	SD (%)	RSD (%)	Concentration (ng/mL)	Recovery (%)	SD (%)	RSD (%)
PA	236.76	101.23	4.40	4.35	78.92	99.80	5.18	5.19
	59.19	106.60	3.10	2.91	19.73	96.57	3.59	3.72
	7.41	104.03	14.02	13.48	2.47	98.73	7.92	8.02
PB	240.75	100.17	3.82	3.81	80.25	103.25	2.24	2.17
	60.18	104.84	3.78	3.61	20.06	98.55	3.00	3.04
	7.53	102.25	12.44	12.17	2.51	104.33	9.70	9.30
PC	230.76	106.84	4.63	4.34	76.92	100.66	1.36	1.35
	57.69	102.27	6.42	6.27	19.23	97.80	1.51	1.54
	7.20	102.81	9.61	9.35	2.40	103.45	10.48	10.14
PE	741.75	104.04	4.86	4.67	247.25	106.51	3.53	3.31
	185.43	108.77	6.49	5.96	61.81	105.03	8.05	7.66
	23.19	105.31	7.67	7.28	3.86	103.25	6.81	6.60
GAS	13,775.01	106.12	5.26	4.96	4,591.67	97.20	4.81	4.94
	3,443.76	109.15	6.48	5.93	1,147.92	104.49	5.16	4.94
	215.25	107.48	8.72	8.12	71.74	105.41	11.52	10.92
4-HBG	204.51	99.76	6.04	6.05	68.17	104.74	2.98	2.85
	51.12	96.23	8.60	8.94	17.04	103.16	8.21	7.95
	6.39	100.56	7.22	7.18	2.13	99.95	7.17	7.18

#### 3.4.5 Stability

Table 6 summarizes the stability results. The RSD of plasma and brain tissue samples after 1 month of storage at −80°C and 3 freeze-thaw cycles at −80°C was less than ± 15.0%. These results suggested that the 6 components were stable in brain tissue and plasma under different conditions (Table 6).

**TABLE 6:** Stability of PA, PB, PC, PE, GAS, and 4-HBG in plasma and brain tissue (n = 6).

Compounds	Plasma					Brain tissue				
	Concentration (ng/mL)	Long-term stability (%)	RSD (%)	Freeze-thaw cycle stability (%)	RSD (%)	Concentration (ng/mL)	Long-term stability (%)	RSD (%)	Freeze-thaw cycle stability (%)	RSD (%)
PA	236.76	104.71	4.31	104.82	3.48	78.92	91.82	8.85	94.25	8.38
	59.19	111.75	3.67	102.52	9.62	19.73	101.5	4.96	94.59	7.55
	7.41	105.99	7.24	104.88	10.07	2.47	104.07	10.06	90.85	11.35
PB	240.75	103.17	4.50	100.87	3.29	80.25	99.56	12.18	99.75	3.46
	60.18	102.25	5.37	106.07	5.93	20.06	103.00	6.61	98.55	3.04
	7.53	108.25	10.57	118.77	9.63	2.51	98.57	11.98	104.33	9.30
PC	230.76	106.7	4.90	106.97	5.15	76.92	102.69	5.59	98.07	1.69
	57.69	104.03	10.28	107.66	10.86	19.23	99.47	4.98	97.80	1.54
	7.2	97.01	11.12	99.69	8.67	2.40	97.93	13.02	95.38	11.24
PE	741.75	107.11	5.85	102.39	5.64	247.25	98.81	9.97	107.52	5.43
	185.43	108.03	4.72	104.69	5.16	61.81	106.91	8.11	105.03	7.66
	23.19	102.85	14.19	94.34	2.53	3.86	106.27	12.50	103.25	6.60
GAS	13,775.01	99.14	12.71	100.22	13.37	4,591.67	101.76	13.20	101.70	14.57
	3,443.76	110.46	11.40	102.08	8.74	1,147.92	96.12	5.63	107.43	4.38
	215.25	106.73	7.69	101.88	5.41	71.74	97.63	9.91	106.77	5.53
4-HBG	204.51	110.04	3.79	102.52	3.70	68.17	100.87	6.51	100.98	5.22
	51.12	106.44	9.94	105.67	3.96	17.04	108.36	10.79	99.96	5.82
	6.39	113.43	6.60	109.36	9.23	2.13	102.77	13.05	100.14	10.95

### 3.5 Pharmacokinetics of GEB extract in normal and MCAO rats

**FIGURE 7:**
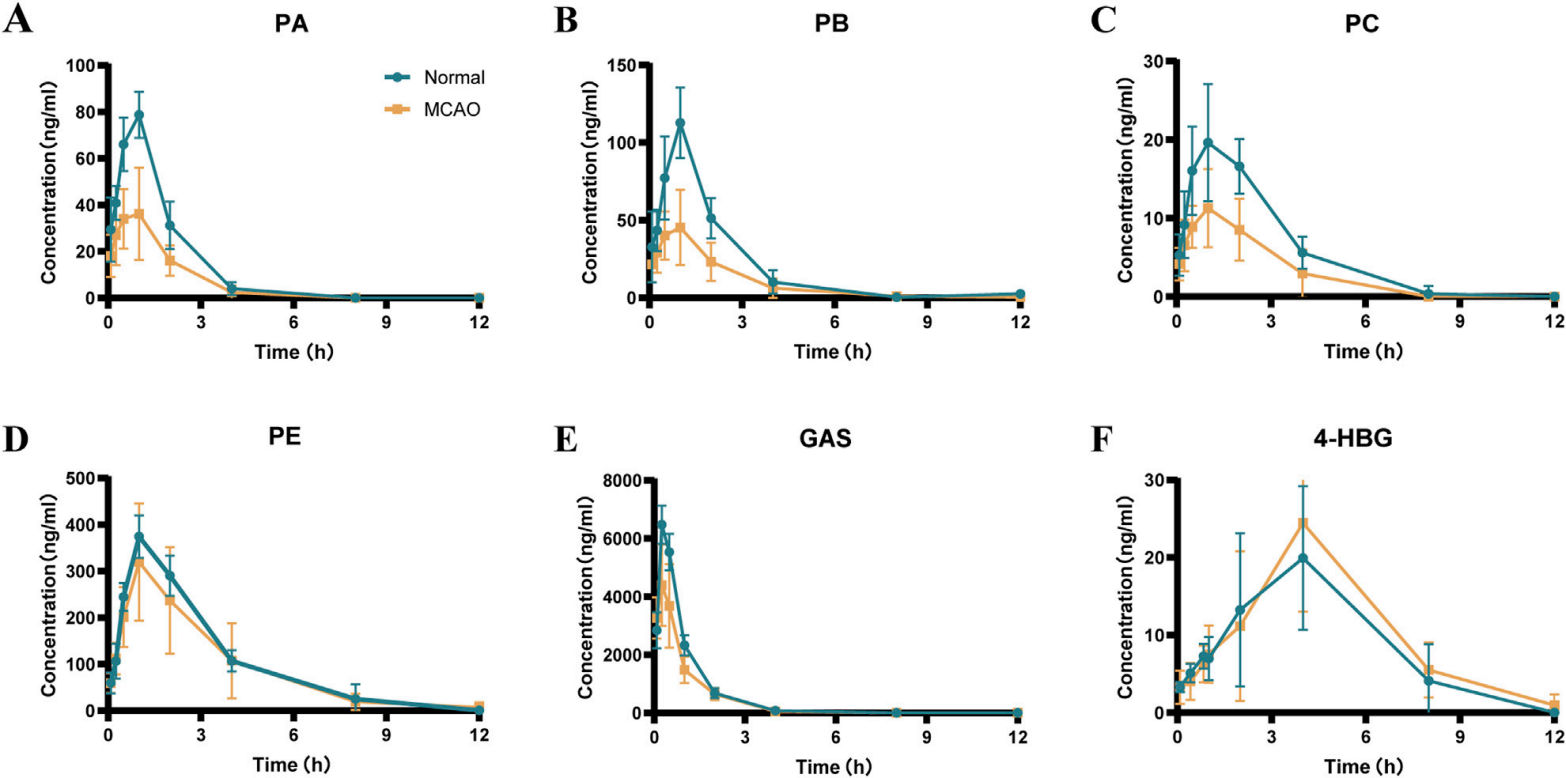
Mean plasma concentration-time curves of the 6 components in normal and MCAO rats after oral administration of GEB extract (mean ± SEM, n = 8). Blue represents normal rats and orange represents MCAO rats. n = 8. **(A)** PA, **(B)** PB, **(C)** PC, **(D)** PE, **(E)** GAS, **(F)** 4-HBG.

Pharmacokinetic profiling of six GEB-derived compounds (PA, PB, PC, PE, GAS, 4-HBG) was conducted via non-compartmental analysis following oral administration (Figure 7; Table 7). GAS, recognized as a principal bioactive constituent, demonstrated the highest systemic exposure in both normal and MCAO rats (AUC_0-t_: 6,620 ± 365 vs. 4,836 ± 791 ng*h/mL; C_max_: 6,620 ± 392 vs. 4,646 ± 1,195 ng/mL, *p* < 0.05), followed by PE, PB, PA, and PC. All compounds exhibited rapid absorption (T_max_ < 2.5 h) and short elimination half-lives (T_1/2_ < 4 h), indicating favorable *in vivo* disposition kinetics. Notably, MCAO rats showed significantly reduced exposure parameters (AUC_0-_ ∞ decreased 26.5%–46.3%; C_max_ declined 29.8%–56.8%, *p* < 0.05) and elevated clearance rates (CL_z/F_ increased 1.4–2.0 fold, *p* < 0.05) for PA, PB, PC, and GAS compared to controls, suggesting ischemia-induced impairment of enterohepatic recirculation and renal reabsorption (Xu et al., 2024)^-^ (Xu et al., 2016). In contrast, PE maintained comparable pharmacokinetic metrics between groups (AUC_0-t_ 1,314 ± 310 vs. 1,148 ± 530 ng h/mL, *p* > 0.05).

**TABLE 7:** Pharmacokinetic parameters of six analytes in normal and MCAO rats after administration of GEB extract at the dosage of 4.8 g/kg (n = 8).

Analytes	Group	Dose	AUC_0-t_	${\text{AUC}}_{0-\infty }$	MRT_0-t_	C_max_	T_max_	T_1/2_	CL_z/F_	V_z/F_
		(mg/kg)	(ng*h/mL)	(ng*h/mL)	(h)	(ng/mL)	(h)	(h)	(L/h/kg)	(L/kg)
PA	Normal	73.30	147.5 ± 21.9	152.8 ± 23.5	1.26 ± 0.09	79.8 ± 9.8	0.94 ± 0.18	0.74 ± 0.10	489.2 ± 69.4	518.7 ± 88.3
	MCAO		75.4 ± 29.3^***^	85.9 ± 25.0^***^	1.33 ± 0.27	41.5 ± 18.5^***^	0.78 ± 0.31	1.41 ± 1.75	908.4 ± 234.5^***^	1706.9 ± 1891.6^*^
PB	Normal	37.09	215.2 ± 37.4	230.4 ± 50.9	1.42 ± 0.13	112.8 ± 22.8	1.00 ± 0.00	1.01 ± 0.42	167.3 ± 34.2	237.9 ± 98.4
	MCAO		108.0 ± 48.8^***^	123.7 ± 38.8^***^	1.63 ± 0.53	48.8 ± 22.9^***^	0.72 ± 0.31^*^	2.16 ± 2.76	329.8 ± 105.3^***^	1,034.1 ± 1,468.2
PC	Normal	6.89	56.4 ± 16.2	71.1 ± 18.5	1.78 ± 0.30	21.5 ± 5.8	0.94 ± 0.50	1.84 ± 0.56	104.1 ± 26.7	266.7 ± 78.4
	MCAO		30.1 ± 9.6^***^	41.5 ± 15.7^**^	1.68 ± 0.29	12.1 ± 4.6^***^	1.13 ± 0.35	2.35 ± 2.08	184.7 ± 64.4^**^	543.6 ± 442.8
PE	Normal	41.36	1,214.8 ± 139.5	1,314.3 ± 310.2	2.41 ± 0.33	375.1 ± 45.5	1.13 ± 0.35	1.82 ± 1.13	32. 6 ± 6.0	78.6 ± 24.2
	MCAO		1,107.5 ± 517.6	1,147.5 ± 530.4	2.68 ± 0.76	324.9 ± 129.0	1.25 ± 0.46	1.95 ± 1.03	41.8 ± 15.9	113.9 ± 64.2
GAS	Normal	15.40	6,619.9 ± 365.1	6,685.6 ± 386.2	0.83 ± 0.06	6,620.2 ± 391.8	0.31 ± 0.12	0.60 ± 0.07	2.3 ± 0.1	2.0 ± 0.2
	MCAO		4,835.6 ± 790.9^***^	4,914.0 ± 728.3^***^	0.88 ± 0.20	4,646.2 ± 1,195.9^***^	0.27 ± 0.16	0.58 ± 0.29	3.2 ± 0.5^***^	2.7 ± 1.6
4-HBG	Normal	7.22	112.6 ± 37.7	119.9 ± 37.0	4.18 ± 0.51	24.1 ± 8.4	3.50 ± 0.93	2.27 ± 1.14	65.2 ± 18.9	212.2 ± 102.2
	MCAO		124.8 ± 51.3	129.1 ± 52.8	4.37 ± 0.50	24.6 ± 11.2	3.75 ± 0.71	2.03 ± 0.24	63.2 ± 22.0	186.8 ± 73.0

Notes: * means P < 0.05, ** means P < 0.01, *** means P < 0.005.

The novel glutathione conjugate 4-HBG displayed distinct absorption kinetics (Tmax 2.3 ± 1.1 vs. 2.03 ± 0.2 h) without inter-group variation (AUC_0-t_ 120 ± 37 vs. 129 ± 53 ng*h/mL, *p* > 0.05). This delayed absorption profile aligns with its structural complexity as a p-hydroxybenzyl-glutathione adduct. Mechanistically, 4-HBG’s neuroprotective efficacy against glutamate excitotoxicity has been attributed to competitive inhibition of kainate receptor binding (Andersson et al., 1995), corroborating its therapeutic potential despite moderate systemic exposure.

### 3.6 Brain tissue distribution of GEB extract in normal and MCAO rats

**FIGURE 8:**
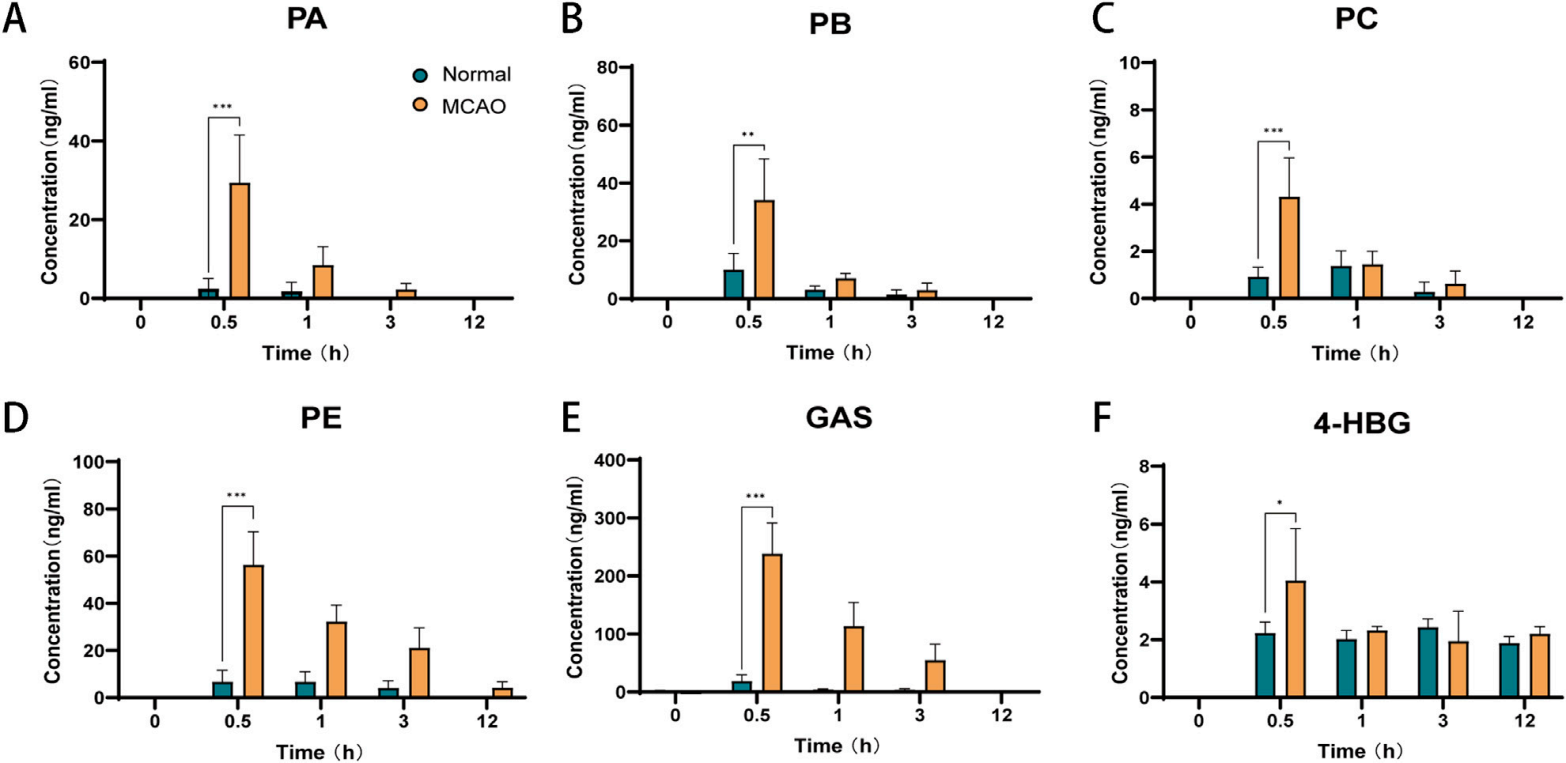
Brain tissue distribution of six components of GEB extract after oral administration in normal and cerebral infarction rats (mean ± SEM, n = 6). Blue represents normal rats and orange represents MCAO rats. ^*^ *P* < 0.05, ^**^ *P* < 0.01, ^***^ *P* < 0.005. n = 6. **(A)** PA, **(B)** PB, **(C)** PC, **(D)** PE, **(E)** GAS, **(F)** 4-HBG.

Cerebral distribution analysis revealed distinct patterns of six GEB-derived compounds (PA, PB, PC, PE, GAS, 4-HBG) between normal and MCAO rats following oral administration (Figure 8). In normophysiological conditions, GAS exhibited limited BBB penetrability (<40 ng/mL cerebral concentration), with all compounds peaking within 0.5–1 h post-dosing. In contrast, ischemic pathophysiology significantly enhanced BBB permeability, resulting in 1.8–12.9-fold higher cerebral concentrations of all analytes in MCAO rats compared to controls (p < 0.01). Notably, sustained cerebral retention was observed in MCAO cohorts, with measurable PE (4.25 ± 2.46 ng/g) concentrations persisting at 12 h—a timepoint when these components were undetectable in normal brains (<LLOQ). This ischemia-facilitated cerebral sequestration of bioactive constituents provides direct pharmacological evidence supporting their roles as critical mediators of GEB’s therapeutic effects against ischemic injury (Wang et al., 2008).

### 3.7 Xenobiotic metabolite distribution in normal and MCAO rats

Pharmacokinetic data showed that both PA and GAS exhibited measurable exposure in rats, while the concentration of their terminal hydrolysis product HBA was very low. To determine whether this phenomenon resulted from the slow metabolic conversion of GAS to HBA or rapid metabolism of HBA below detectable limits, this study systematically investigated the complete metabolic pathway from PA to HBA. Furthermore, given the pivotal role of xenobiotic metabolites in drug metabolism, elucidating their dynamics is crucial for understanding the pharmacological, toxicological, and physiological effects of drugs *in vivo.*

Exogenous compounds are absorbed into the body and undergo stepwise metabolic pathways that produce precise changes in mass numbers. Theoretically, the metabolites of a compound can be predicted because most metabolic reactions, such as reduction, oxidation, deglycosylation, hydroxylation, and sulfonation coupling, are well-known (Gong et al., 2012). This investigation evaluated all possible metabolic processes based on the characteristics of the series of compounds that undergo stepwise hydrolysis from PA to HBA (4-HBG is not included because it is not obtained through stepwise hydrolysis of PA). Furthermore, a xenobiotic metabolic patterns database of all the compounds contained in PA to HBA was established in sequence (Supplementary Table S4).

**FIGURE 9:**
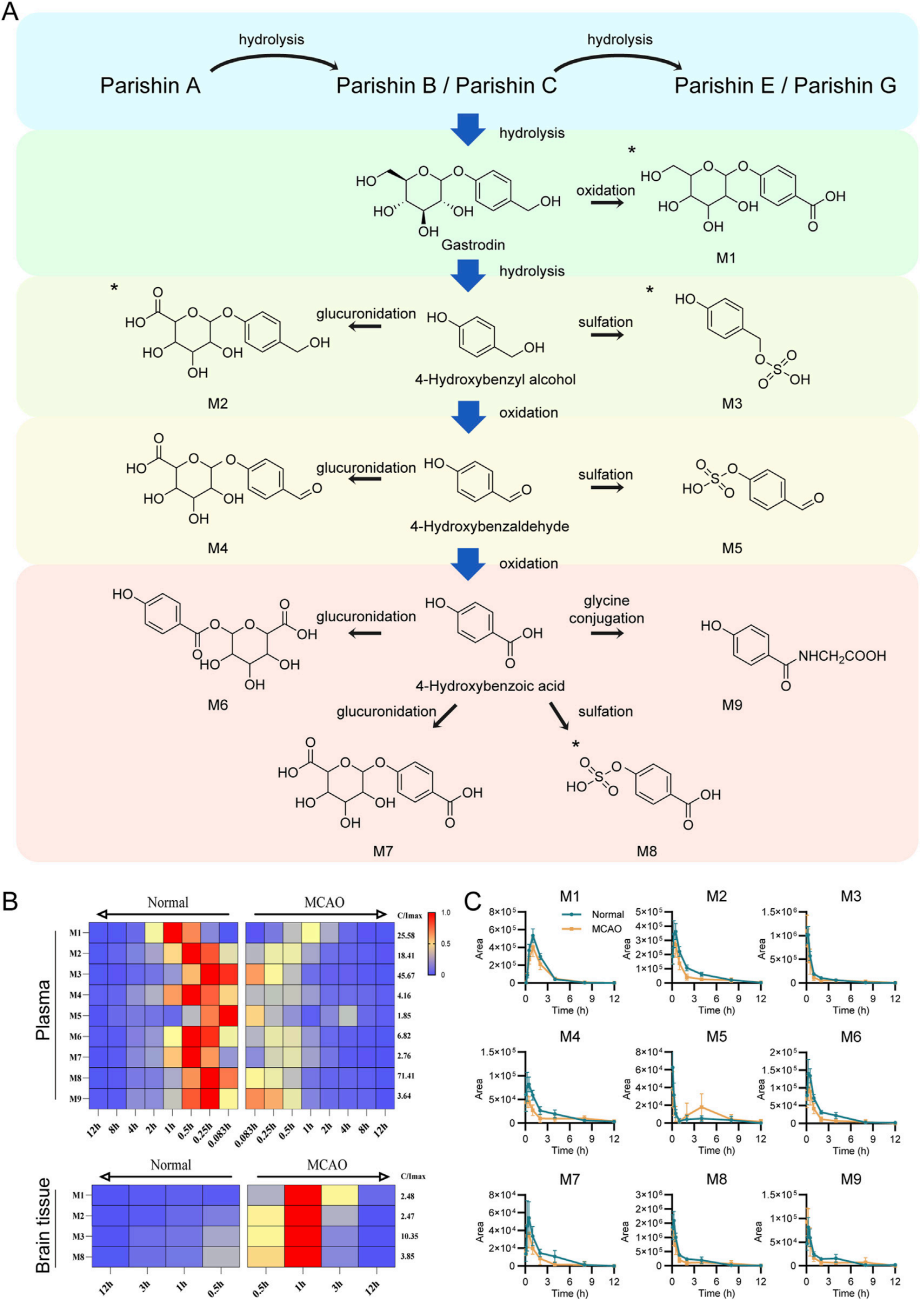
Identification of validated xenobiotic metabolites in Normal and MCAO model rats after oral administration of GEB extract. **(A)** Sequential hydrolysis and metabolic pathways of xenobiotic metabolites from PA to HBA. All metabolites were detected in plasma, with those identified in brain tissue marked by asterisks (*). **(B)** Time-dependent heatmap visualization of xenobiotic metabolites in plasma (n = 8) and brain tissue (n = 6). Data were normalized to the internal standard (IS) and expressed as relative abundance. The maximum value is set to red, the minimum to blue, and intermediate values to yellow (maximum intensity scale shown on the right). Normal rats and MCAO rats are positioned on the left and right sides, respectively. The x-axis utilizes mirrored time scales to facilitate horizontal comparison between groups. The y-axis displays the distinct types of identified xenobiotic metabolites. **(C)** Comparative peak area profiles of nine xenobiotic metabolites in plasma between Normal and MCAO groups.

Plasma and brain tissue samples were analyzed by UPLC-QTOF-MS in negative ion mode to obtain detailed mass spectral data. Peak extraction was carried out using MassHunter Profinder software B.10.0, and data were saved in as. csv format. The PCA analysis revealed certain regularity in the changes in the rat’s plasma components (Supplementary Figure S1). Furthermore, using the R software, metabolic patterns database and digitized mass spectrometry data were matched to obtain the metabolic data of the compounds (R code is provided in the Supplementary Material). Using MassHunter qualitative analysis software, nine exogenous metabolites were identified by correlating fragment ion m/z values with fragmentation patterns, four of which were detected in brain tissue (The EIC plots of the administered and blank samples are shown in Supplementary Figures S2–S3. Retention times of potential metabolites, fragment ions, and proposed fragmentation pathways are shown in Supplementary Figures S4–S12). Figure 9A shows the specific xenobiotic metabolites detected in rat plasma after stepwise hydrolysis from PA to HBA.

It was observed that parishin-type compounds metabolism mainly includes hydrolysis and is eventually excreted as GAS or HBA metabolites. Given the minimal HBA levels alongside substantial xenobiotic metabolites detected in rats, we hypothesize that partial hydrolysis of GAS to HBA may occur, but HBA is rapidly cleared *in vivo*, resulting in concentrations below the limit of detection. Due to pathological changes in the metabolism of MCAO rats, the relative content of xenobiotic metabolites was lower, and the peak value and metabolic rate of xenobiotic metabolites were faster relative to the normal rats (Figures 9B,C). In the MCAO rat’s brain tissue, the peak areas of 4 xenobiotic metabolites were higher than those in normal rats (Supplementary Figure S13). This result was consistent with the distribution of the parent products, suggesting that the corresponding xenobiotic metabolites had a considerable distribution in the brain after cerebral ischemia.

## 4 Discussion

Pathophysiological alterations, including dysregulated tissue pH and inflammatory mediator infiltration, profoundly modify drug disposition through dual mechanisms: (1) physicochemical modulation of blood/tissue barriers affecting membrane permeability; and (2) functional impairment of metabolic enzymes and transport systems. These changes collectively perturb drug absorption, distribution, metabolism, and excretion (ADME) processes. Under cerebral ischemia-reperfusion conditions, drug distribution patterns are significantly altered, whether due to compromised gastrointestinal absorption capacity, impaired renal metabolic function, or altered permeability resulting from cerebral edema. The spatiotemporal distribution of bioactive compounds in target organs directly determines their pharmacological effects. Therefore, investigating the pharmacokinetic differences of the GEB extract between normal and cerebral ischemia rat models is crucial for elucidating its mechanism of action and optimizing its clinical translation.

Building upon the methodological foundation established for characterizing GEB’s constituents and metabolites, and given the well-documented neuroprotective efficacy of GEB in both traditional use and modern pharmacology, it is imperative to understand the pharmacokinetic behavior of its key bioactive components under pathological conditions to elucidate their contribution to the overall therapeutic outcome. Our previous studies demonstrated that *Gastrodia elata* extract exhibits therapeutic effects against CIRI, with the GEB-H showing significantly superior efficacy to the GEB-L; based on these findings, this study developed a sensitive UPLC-Q-TOF-MS method for detailed characterization of GEB extracts, identifying 53 constituents and prioritizing six bioactive parent compounds (PA, PB, PC, PE, GAS, 4-HBG) for pharmacokinetic evaluation. A validated UPLC-QQQ-MS method enabled precise quantification of these analytes in plasma and brain tissues over 12 h post-administration, with all six target analytes achieving quantitation at ng/mL levels in plasma and ng/g levels in brain tissue. Notably, we pioneered an R-based computational pipeline integrating metabolic pattern prediction (±10 ppm mass accuracy) and spectral matching, successfully identifying nine xenobiotic metabolites—four exhibiting cerebral distribution—thereby establishing a methodological foundation for elucidating GEB’s *in vivo* pharmacological xenobiotic mechanisms.

GEB has been empirically employed in traditional medicine for centuries to treat neurological disorders such as depression and insomnia, with its therapeutic efficacy validated through historical and modern pharmacological studies (Zhou et al., 2024). Contemporary research reveals a complex phytochemical profile comprising multiple bioactive constituents that synergistically mediate neuroprotective effects. GAS exerts anti-apoptotic activity in glutamate-challenged neuronal cells by suppressing the CaMKII/ASK-1/p38 MAPK/p53 signaling cascade, reducing oxidative stress and mitochondrial dysfunction (Jiang et al., 2014). N6-(4-Hydroxybenzyl)adenosine enhances GABAergic neurotransmission through adenosine receptor activation, contributing to sedative-hypnotic effects (Zhang et al., 2012). PC ameliorates depressive behaviors by modulating hypothalamic-pituitary-adrenal axis hyperactivity and neurotransmitter balance (Jiang et al., 2024). HBA preserves neural function under ischemic conditions via NMDA-CREB-BDNF pathway modulation (Xiao et al., 2024). Additionally, polybenzyl ether derivatives such as 2,4-bis(4-hydroxybenzyl)phenol demonstrate potent melatonin receptor agonism, further contributing to sedative-hypnotic efficacy (Chen et al., 2019). While biphenyl compounds were undetected in our extracts—potentially due to geographical or processing variations—the identified bioactive components collectively underpin GEB’s neuropharmacological outcomes (Lai et al., 2016).

The pharmacokinetic characterization revealed six primary bioactive components in systemic circulation following GEB extract administration: PA, PB, PC, PE, GAS, and 4-HBG. Notably, HBA—the terminal hydrolysis product of both parishin and GAS—was excluded from pharmacokinetic analysis due to its subtherapeutic plasma concentrations. This phenomenon is likely primarily attributable to HBA’s rapid metabolic conversion via phase II reactions (sulfation/glucuronidation), as strongly suggested by the abundant HBA-derived metabolites detected in biological matrices. While insufficient precursor availability cannot be entirely ruled out as a potential contributing factor, (Lin et al., 2008),^-^ (Zhang et al., 2008), the observed metabolite abundance indicates that extensive phase II metabolism is a dominant driver of the low circulating HBA concentrations. Comparative pharmacokinetic analysis demonstrated distinct disposition patterns among components: GAS exhibited the highest systemic exposure (AUC 0‐∞ and C_max_) but rapid elimination and limited BBB penetration, whereas parishin analogs (PA/PB/PC) showed superior BBB permeability despite lower plasma concentrations, consistent with their elevated lipophilicity. Intriguingly, PE maintained comparable exposure levels between normal and MCAO groups while demonstrating moderate cerebral distribution compared to the superior penetration of parishin analogs (PA/PB/PC) and the limited penetration of GAS. Mechanistically, PA undergoes partial hydrolysis *in vivo* (50% conversion efficiency), releasing an average of 1.5 GAS molecules per parent compound (Tang et al., 2015). This controlled release mechanism, coupled with PA inherent BBB-targeting capability due to amphiphilic structural features, positions PA-class compounds as promising neuropharmacological prodrugs capable of sustained GAS delivery to cerebral compartments.

The metabolic fate of xenobiotics exhibits considerable predictability due to well-characterized biotransformation pathways (Ma et al., 2023). Parent compounds undergo structural modifications during metabolism, manifesting as diagnostic mass shifts detectable through high-resolution mass spectrometry. In this investigation, we constructed a hierarchical metabolic database encompassing sequential hydrolysis products along the PA→HBA transformation axis. Leveraging R-based computational algorithms, we developed a mass defect-driven matching strategy (± 10 ppm tolerance), enabling successful identification of nine xenobiotic metabolites *in vivo*. Furthermore, we propose that parishin, the predominant phenolic compound in *Gastrodia elata*, undergoes stepwise hydrolysis to GAS and HBA, followed by metabolic elimination from the body. This integrative approach demonstrates enhanced capability in resolving complex metabolic networks (Wang et al., 2022), and the above results provide a reference for further studies on efficacy evaluation, quality control, and rational use of drugs.

## 5 Conclusion

This study characterized the pharmacokinetics and cerebral distribution of bioactive compounds from GEB in CIRI. We identified 53 constituents, with six parent compounds (PA, PB, PC, PE, GAS, 4-HBG) achieving systemic and brain exposure. CIRI significantly increased BBB permeability, leading to markedly enhanced cerebral accumulation of all analytes despite reduced plasma levels for most, suggesting their role in GEB’s efficacy.

We innovatively mapped the metabolic pathway from PA to HBA and generated a precise theoretical xenobiotic metabolite database using R programming. This identified nine phase I/II metabolites, four of which showed enhanced cerebral distribution in ischemia. This is the first systematic elucidation of GEB’s *in vivo* metabolic fate, detailing the transformation from parishin-type precursors to terminal phenolic metabolites.

These findings establish the pharmacokinetic and metabolic basis for GEB’s therapeutic effects against CIRI. The identified parent compounds and metabolites collectively form GEB’s pharmacodynamic substance basis. The developed methodology provides a robust framework for herbal medicine quality control and metabolic research, laying essential groundwork for further development of GEB-based therapies.

## Data Availability

The original contributions presented in the study are included in the article/Supplementary Material, further inquiries can be directed to the corresponding authors.

## Abbreviations

AUC, Area under curve; CE, Collision energy; CL_z/F_: Clearance rectified by bioavailability; EIC, Extracted ion chromatogram; GAS, Gastrodin; HBA, 4-Hydroxybenzyl alcohol; IS, Internal standard; LOD, Limit of detection; LLOQ, Lower limit of quantitation; MCAO, Middle Cerebral Artery Occlusion; mNSS, Modified neurological severity score; MRT, Mean resident time; NMDP, Nimodipine; PA, Parishin A; PB, Parishin B; PC, Parishin C; PE, Parishin E; T_max_, Time to reach the maximum concentration; T_1/2_, Elimination half-life; V_z/F_, Apparent volume of distribution rectified by bioavailability; 4-HBG, S-(4-Hydroxybenzyl) Glutathione.
