# Supplementary material for: Pharmacokinetics and brain tissue distribution of Gastrodia elata extract in normal and cerebral ischemic rats: a comparative study
Source: Front Pharmacol. 2025 Jul 31;16:1624576. doi: 10.3389/fphar.2025.1624576 (PMC12350473; doi:10.3389/fphar.2025.1624576)
Supplement: Supplementary file 1 [file Supplementaryfile1.docx]

*Supplemental Tables, Figures and R code*

**Pharmacokinetics and brain tissue distribution of Gastrodia elata extract in normal and cerebral ischemic rats: A comparative study**

**Supplemental File:** 4 Tables, 13 Figures and 5 *R* code

**Supplementary Table 1** Optimized dynamic MRM parameters for 7 components and internal standard (IS)

| **Compounds** | **Precursor ion (*m/z*)** | **Product ion (*m/z*)** | **Dwell time (ms)** | **Q1 pre-bias (V)** | **Collision energy (V)** | **Q3 pre-bias (V)** | **RT/min** |
| --- | --- | --- | --- | --- | --- | --- | --- |
| PA | 995 | 727.45 | 15 | 22 | 28 | 26 | 11.05 |
| PB | 727.2 | 161.3 | 15 | 26 | 33 | 14 | 10.09 |
| PC | 727.2 | 161.3 | 15 | 26 | 33 | 14 | 10.31 |
| PE | 459 | 111.1 | 15 | 12 | 26 | 10 | 9.09 |
| GAS | 330.9 | 45.1 | 15 | 11 | 24 | 13 | 4.02 |
| 4-HBG | 412.1 | 306.25 | 15 | 14 | 16 | 20 | 9.18 |
| Bergenin（IS） | 327.15 | 192.25 | 15 | 11 | 25 | 11 | 9.22 |

**Supplementary Table 2** Concentration of reference substance in high, medium and low QC solutions in plasma and brain tissue homogenate

| Sample | Concentration | PA | PB | PC | PE | GAS | 4-HBG |
| --- | --- | --- | --- | --- | --- | --- | --- |
| Plasma | H | 236.75 | 240.75 | 230.75 | 741.75 | 13775.00 | 204.50 |
|  | M | 59.19 | 60.19 | 57.69 | 185.44 | 3443.75 | 51.13 |
|  | L | 7.40 | 7.52 | 7.21 | 11.59 | 215.23 | 6.39 |
| Brain tissue | H | 78.92 | 80.25 | 76.92 | 247.25 | 4591.67 | 68.17 |
|  | M | 19.73 | 20.06 | 19.23 | 61.81 | 1147.92 | 17.04 |
|  | L | 2.47 | 2.51 | 2.40 | 3.86 | 71.74 | 2.13 |

**Supplementary Table 3** Concentrations of prepared plasma and brain tissue calibration standards

| Sample | NO. | PA | PB | PC | PE | GAS | 4-HBG |
| --- | --- | --- | --- | --- | --- | --- | --- |
| Plasma | A1 | 1.85 | 1.88 | 1.80 | 1.16 | 21.52 | 1.60 |
|  | A2 | 7.40 | 7.52 | 7.21 | 11.59 | 215.23 | 6.39 |
|  | A3 | 29.59 | 30.09 | 28.84 | 92.72 | 1721.88 | 25.56 |
|  | A4 | 59.19 | 60.19 | 57.69 | 185.44 | 3443.75 | 51.13 |
|  | A5 | 118.38 | 120.38 | 115.38 | 370.88 | 6887.50 | 102.25 |
|  | A6 | 236.75 | 240.75 | 230.75 | 741.75 | 13775.00 | 204.50 |
| Brain tissue | A1 | 0.62 | 0.63 | 0.60 | 0.39 | 7.17 | 0.53 |
|  | A2 | 2.47 | 2.51 | 2.40 | 3.86 | 71.74 | 2.13 |
|  | A3 | 9.86 | 10.03 | 9.61 | 30.91 | 573.96 | 8.52 |
|  | A4 | 19.73 | 20.06 | 19.23 | 61.81 | 1147.92 | 17.04 |
|  | A5 | 39.46 | 40.13 | 38.46 | 123.63 | 2295.83 | 34.08 |
|  | A6 | 78.92 | 80.25 | 76.92 | 247.25 | 4591.67 | 68.17 |

**Supplementary Table 4** In vivo predicted conversion pathways of Parishin A, Parishin B/C, Parishin E/G, GAS, and HBA

| **Prototype** | **Prototype accurate mass** | **Description** | **Metabolic reaction** | **△*m/z*** | **Parameter** |
| --- | --- | --- | --- | --- | --- |
| Parishin A | 996.31105 | Oxidation | -H2+O | +13.9793 | a |
| Parishin A | 996.31105 | Hydroxylation | +O | +15.9949 | b |
| Parishin A | 996.31105 | Glucosidation | +C6H10O5 | +162.0528 | c |
| Parishin A | 996.31105 | Sulfoconjugation | +SO3 | +79.9568 | d |
| Parishin A | 996.31105 | Glucuronide conjugation | +C6H8O6 | +176.0321 | e |
| Parishin A | 996.31105 | Glucosidation | +C6H10O5 | +162.0528 | f |
| Parishin B/C | 728.21636 | Glucuronide conjugation | +C6H8O6 | +176.0321 | a |
| Parishin B/C | 728.21636 | Oxidation | -H2+O | +13.9793 | b |
| Parishin B/C | 728.21636 | Hydroxylation | +O | +15.9949 | c |
| Parishin B/C | 728.21636 | Dehydration | -H2O | -18.0106 | d |
| Parishin B/C | 728.21636 | Glucosidation | +C6H10O5 | +162.0528 | e |
| Parishin B/C | 728.21636 | Sulfoconjugation | +SO3 | +79.9568 | f |
| Parishin B/C | 728.21636 | Deglycosylation | -C6H10O5 | -162.0528 | g |
| Parishin B/C | 728.21636 | Decarboxylation | -CO2 | -43.9898 | h |
| Parishin B/C | 728.21636 | Glycine conjugation | +CzHзNO | +57.021464 | i |
| Parishin B/C | 728.21636 | Cysteine conjugation | +C3H5NOS | +103.0092 | j |
| Parishin B/C | 728.21636 | Taurine conjugation | +C2H5NO2S | +107.004101 | k |
| Parishin B/C | 728.21636 | Glutamine conjugation | +C5H7NO3 | +129.042594 | l |
| Parishin B/C | 728.21636 | Carnitine coniugation | +C7H14NO2 | +144.102454 | m |
| Parishin B/C | 728.21636 | S-N-acetylcysteine conjugation | +C5H7NO2S | +145.0198 | n |
| Parishin B/C | 728.21636 | S-acyl-glutathione conjugates | +C10H15N3O5S | +289.073244 | o |
| Parishin E/G | 460.12167 | Glucuronide conjugation | +C6H8O6 | +176.0321 | a |
| Parishin E/G | 460.12167 | Oxidation | -H2+O | +13.9793 | b |
| Parishin E/G | 460.12167 | Hydroxylation | +O | +15.9949 | c |
| Parishin E/G | 460.12167 | Dehydration | -H2O | -18.0106 | d |
| Parishin E/G | 460.12167 | Glucosidation | +C6H10O5 | +162.0528 | e |
| Parishin E/G | 460.12167 | Sulfoconjugation | +SO3 | +79.9568 | f |
| Parishin E/G | 460.12167 | Deglycosylation | -C6H10O5 | -162.0528 | g |
| Parishin E/G | 460.12167 | Decarboxylation | -CO2 | -43.9898 | h |
| Parishin E/G | 460.12167 | Glycine conjugation | +C2H5NO | +57.021464 | i |
| Parishin E/G | 460.12167 | Cysteine conjugation | +C3H5NOS | +103.009186 | j |
| Parishin E/G | 460.12167 | Taurine conjugation | +C2H5NO2S | +107.004101 | k |
| Parishin E/G | 460.12167 | Glutamine conjugation | +C5H7NO3 | +129.042594 | l |
| Parishin E/G | 460.12167 | Carnitine coniugation | +C7H14NO2 | +144.102454 | m |
| Parishin E/G | 460.12167 | S-N-acetylcysteine conjugation | +C5H7NO2S | +145.019751 | n |
| Parishin E/G | 460.12167 | S-acyl-glutathione conjugates | +C10H15N3O5S | +289.073244 | o |
| Gastrodin | 286.1053 | Glucuronide conjugation | +C6H8O6 | +176.0321 | a |
| Gastrodin | 286.1053 | Oxidation | -H2 | -2.0157 | b |
| Gastrodin | 286.1053 | Oxidation- | -H2+O | +13.9793 | c |
| Gastrodin | 286.1053 | Hydroxylation | +O | +15.9949 | d |
| Gastrodin | 286.1053 | Glucosidation | +C6H10O5 | +162.0528 | e |
| Gastrodin | 286.1053 | Sulfoconjugation | +SO3 | +79.9568 | f |
| Gastrodin | 302.100215 | Oxidation and Glycine conjugation | +C2H5NO | +57.021464 | g |
| Gastrodin | 302.100215 | Oxidation and Cysteine conjugation | +C3H5NOS | +103.0092 | h |
| Gastrodin | 302.100215 | Oxidation and Taurine conjugation | +C2H5NO2S | +107.004101 | i |
| Gastrodin | 302.100215 | Oxidation and Glutamine conjugation | +C5H7NO3 | +129.042594 | j |
| Gastrodin | 302.100215 | Oxidation and Carnitine coniugation | +C7H14NO2 | +144.102454 | k |
| Gastrodin | 302.100215 | Oxidation and S-N-acetylcysteine conjugation | +C5H7NO2S | +145.019751 | l |
| Gastrodin | 302.100215 | Oxidation and S-acyl-glutathione conjugates | +C10H15N3O5S | +289.073244 | m |
| p-Hydroxybenzyl Alcohol | 124.0524 | Glucuronide conjugation | +C6H8O6 | +176.0321 | a |
| p-Hydroxybenzyl Alcohol | 124.0524 | Oxidation | -H2 | -2.01565 | b |
| p-Hydroxybenzyl Alcohol | 124.0524 | Oxidation | -H2+O | +13.9793 | c |
| p-Hydroxybenzyl Alcohol | 124.0524 | Hydroxylation | +O | +15.9949 | d |
| p-Hydroxybenzyl Alcohol | 124.0524 | Sulfoconjugation | +SO3 | +79.9568 | e |
| p-Hydroxybenzyl Alcohol | 124.0524 | Oxidation and Glycine conjugation | +C2H5NO | +57.021464 | f |
| p-Hydroxybenzyl Alcohol | 124.0524 | Oxidation and Cysteine conjugation | +C3H5NOS | +103.009186 | g |
| p-Hydroxybenzyl Alcohol | 124.0524 | Oxidation and Taurine conjugation | +C2H5NO2S | +107.004101 | h |
| p-Hydroxybenzyl Alcohol | 124.0524 | Oxidation and Glutamine conjugation | +C5H7NO3 | +129.042594 | i |
| p-Hydroxybenzyl Alcohol | 124.0524 | Oxidation and Carnitine coniugation | +C7H14NO2 | +144.102454 | j |
| p-Hydroxybenzyl Alcohol | 124.0524 | Oxidation and S-N-acetylcysteine conjugation | +C5H7NO2S | +145.019751 | k |
| p-Hydroxybenzyl Alcohol | 124.0524 | Oxidation and S-acyl-glutathione conjugates | +C10H15N3O5S | +289.073244 | l |

**Supplementary Figure 1** PCA analysis of plasma composition over time in rats, A normal rat, Bmcao rat


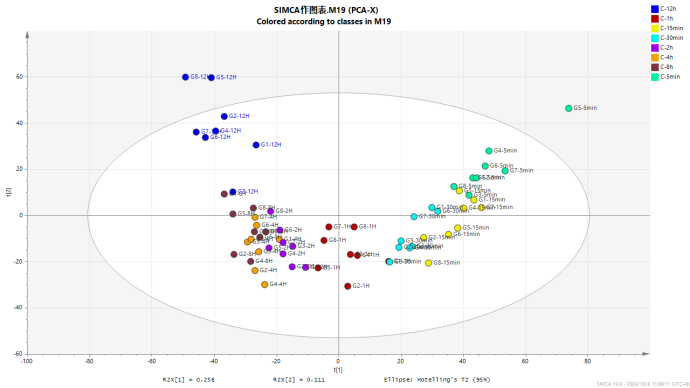

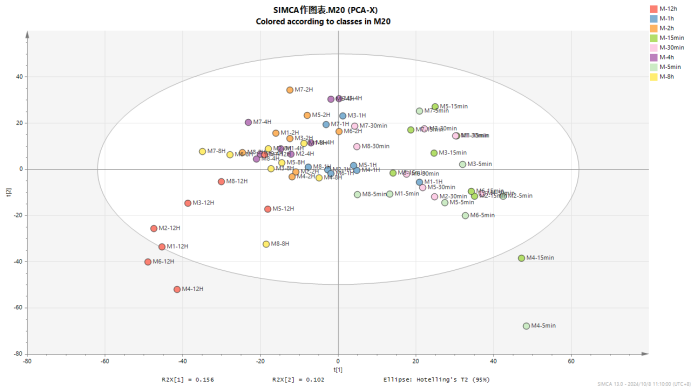

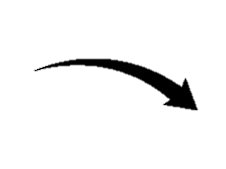

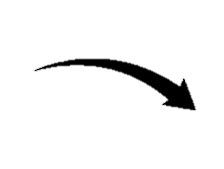


A

B

**Supplementary Figure 2**. EIC plot of metabolites in rat plasma compared with blank plasma 15 min after drug administration.


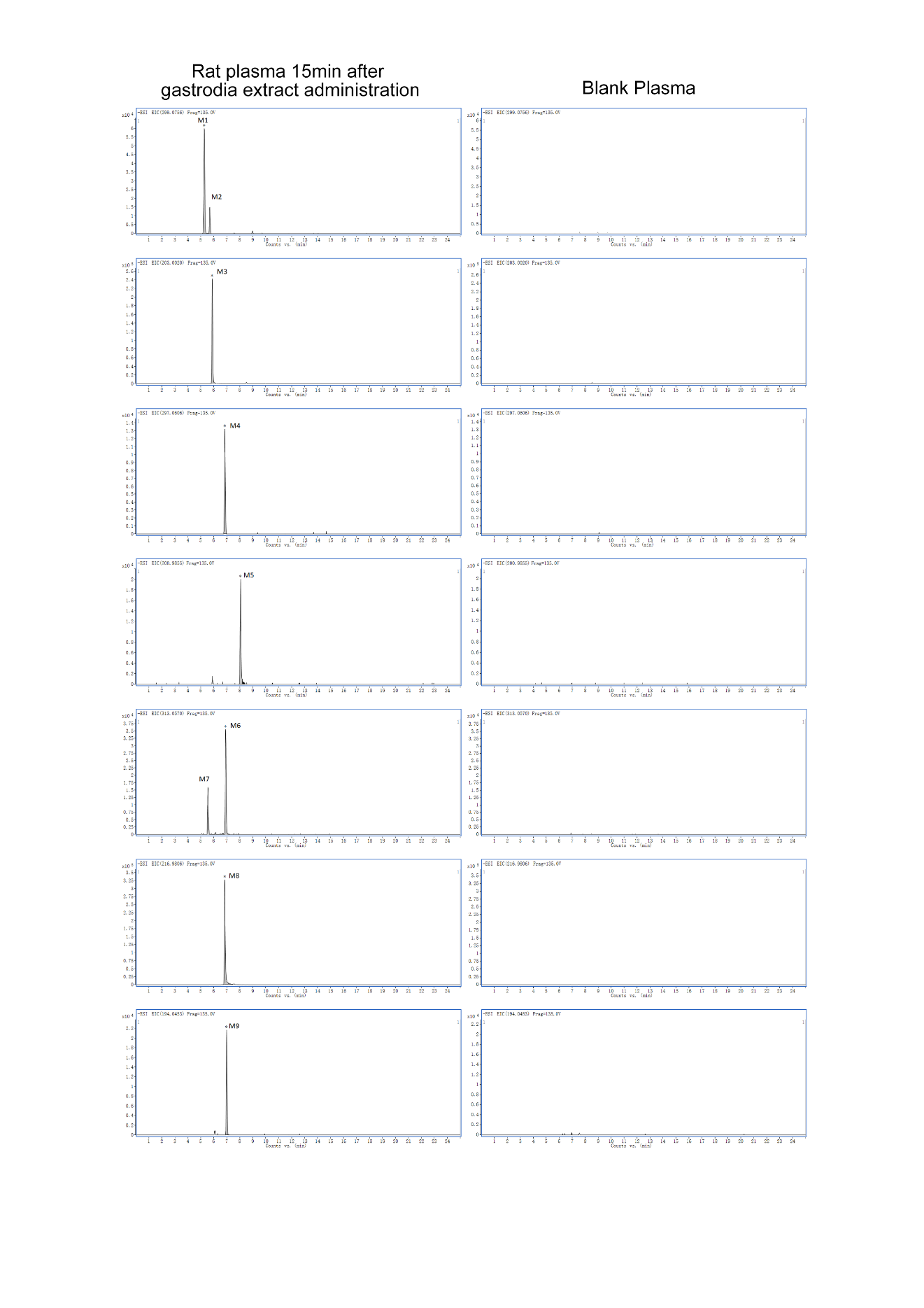


**Supplementary Figure 3**. EIC plot of rat brain tissue versus blank brain tissue 1 h after drug administration, metabolites.


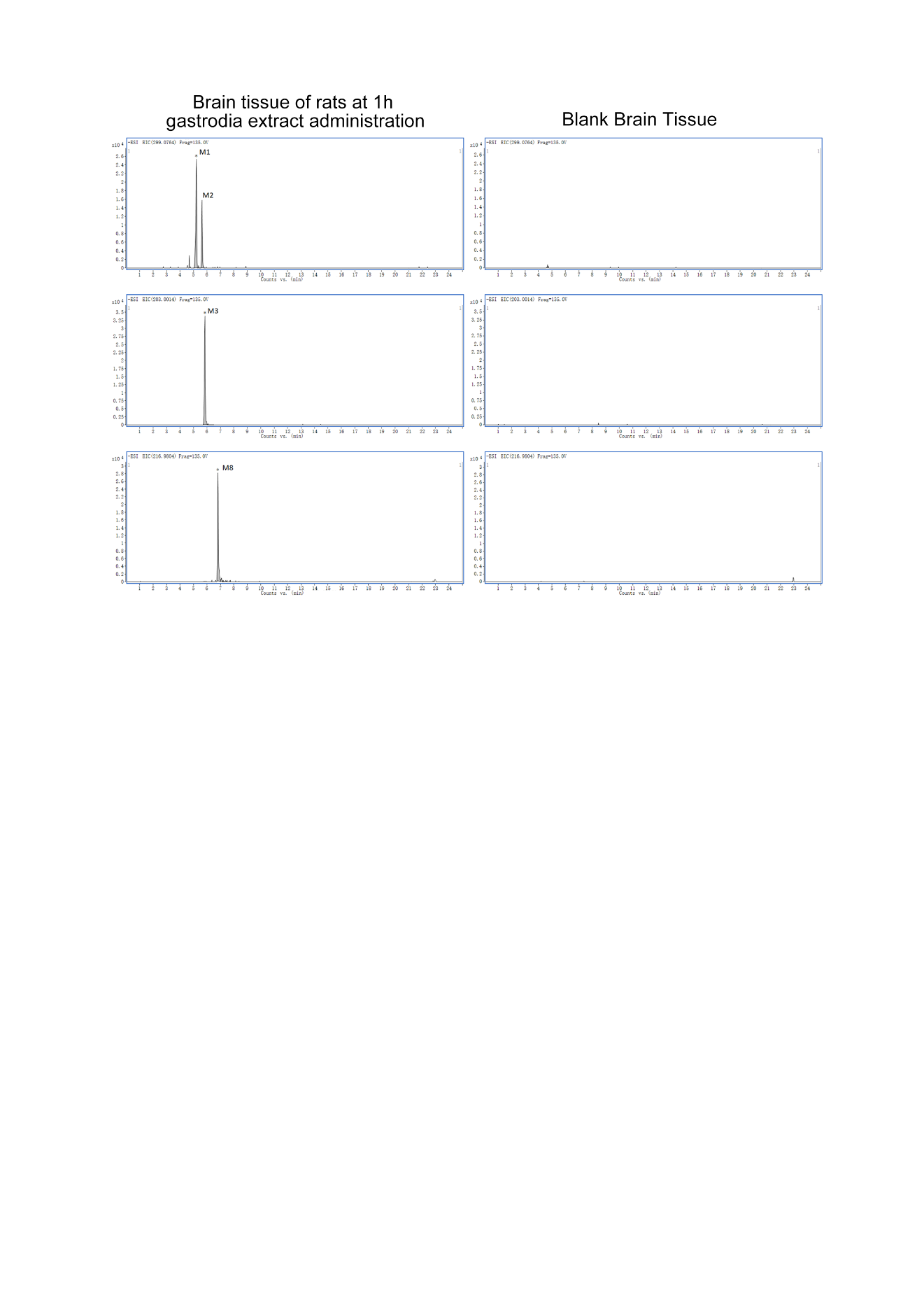


**The MS and MS/MS of 9 metabolites in rats (Figure S4–S13)**

**Supplementary Figure 4**. MS and MS/MS spectra of metabolite (M1) in negative ESI mode and the proposed fragmentation pathway.


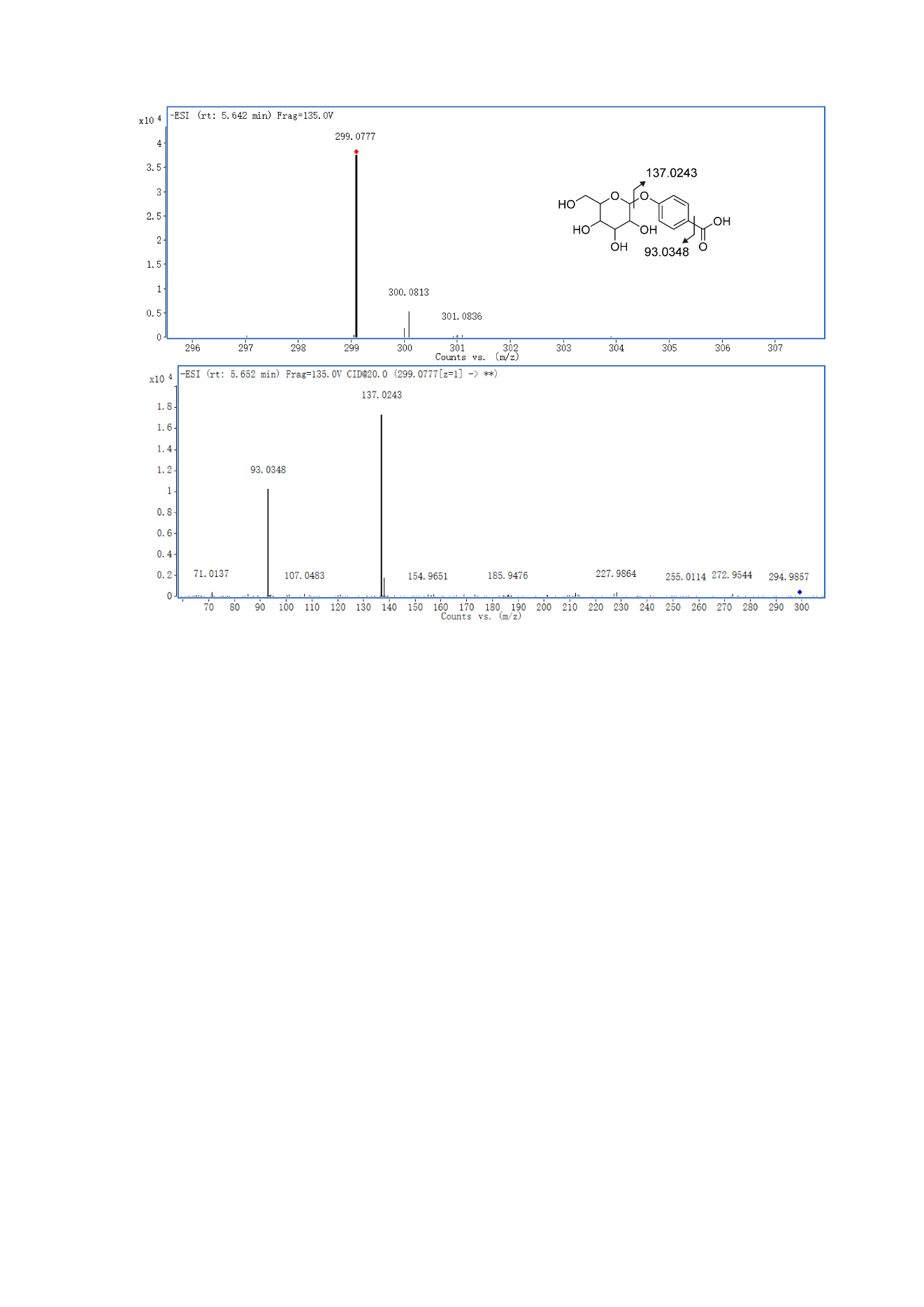


M1 at 5.642 min exhibited a [M - H]^-^ ion at *m/z* 299.0777, which was 14 Da higher than that of GAS. The fragment ions at *m/z* 137.0243 and 93.0348 indicated the presence of a *p*-hydroxybenzoic acid moiety. Additionally, the precursor generated a fragment ion at *m/z* 137.0248 through the neutral loss of a glucose unit (−162.05 Da). Based on these observations, M1 was identified as an oxidation product of GAS, designated as GAS -COOH (where the alcohol hydroxyl group of GAS is oxidized to a carboxyl group).

**Supplementary Figure 5**. MS and MS/MS spectra of metabolite (M2) in negative ESI mode and the proposed fragmentation pathway.


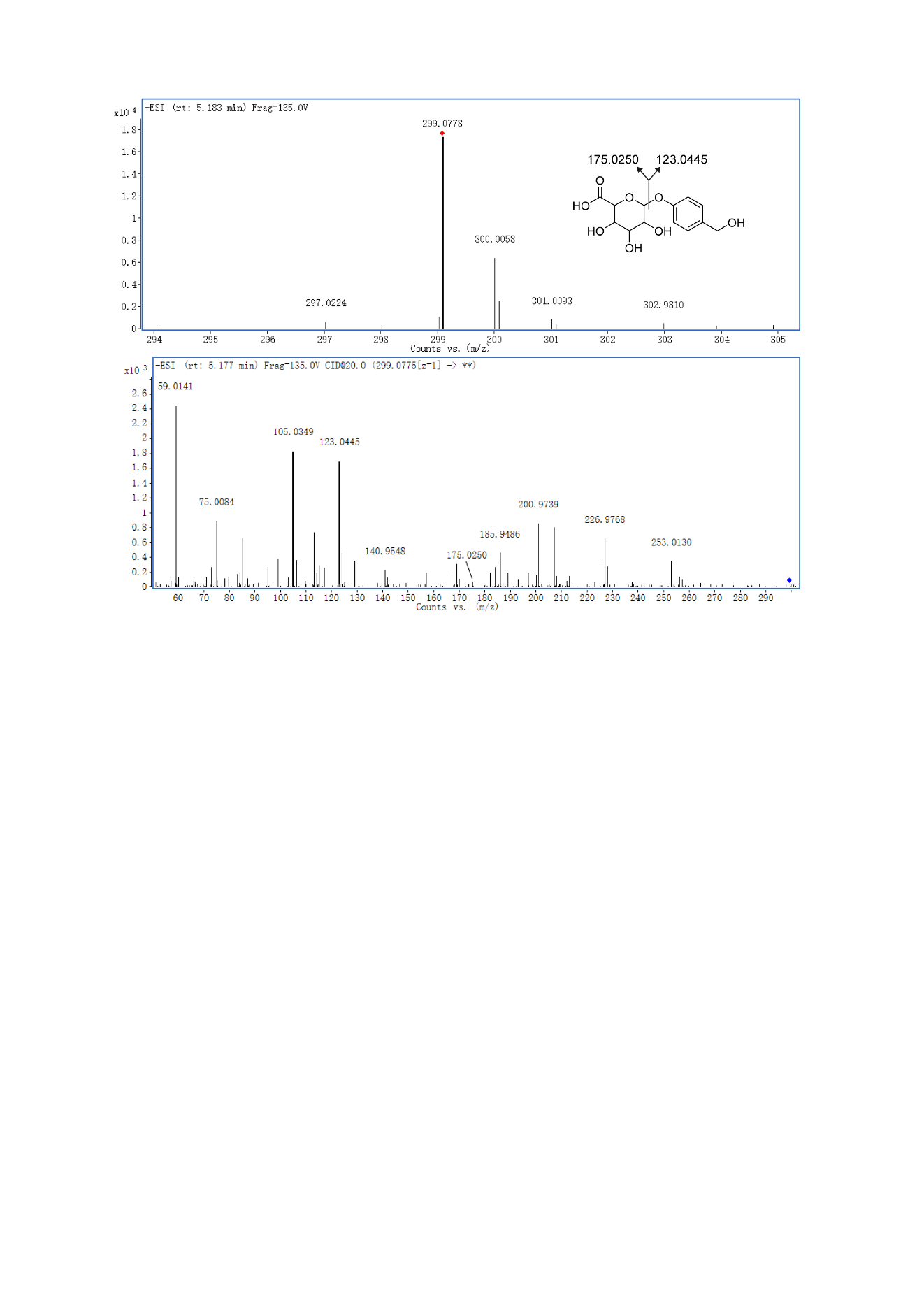


M2, eluting at 5.183 min, shared the same precursor ion ([M - H]^-^ at *m/z* 299.0778) as M1 (retention time: 5.642 min). However, M2 generated distinct fragment ions at *m/z* 123.0445 and 105.0349, which were consistent with the presence of a HBA moiety. Additionally, the fragment ion at *m/z* 175.0250 was identified as a diagnostic marker for glucuronide-conjugated metabolites. Based on this evidence, M2 was assigned as the glucuronide conjugate of HBA.

**Supplementary Figure 6**. MS and MS/MS spectra of metabolite (M3) in negative ESI mode and the proposed fragmentation pathway.


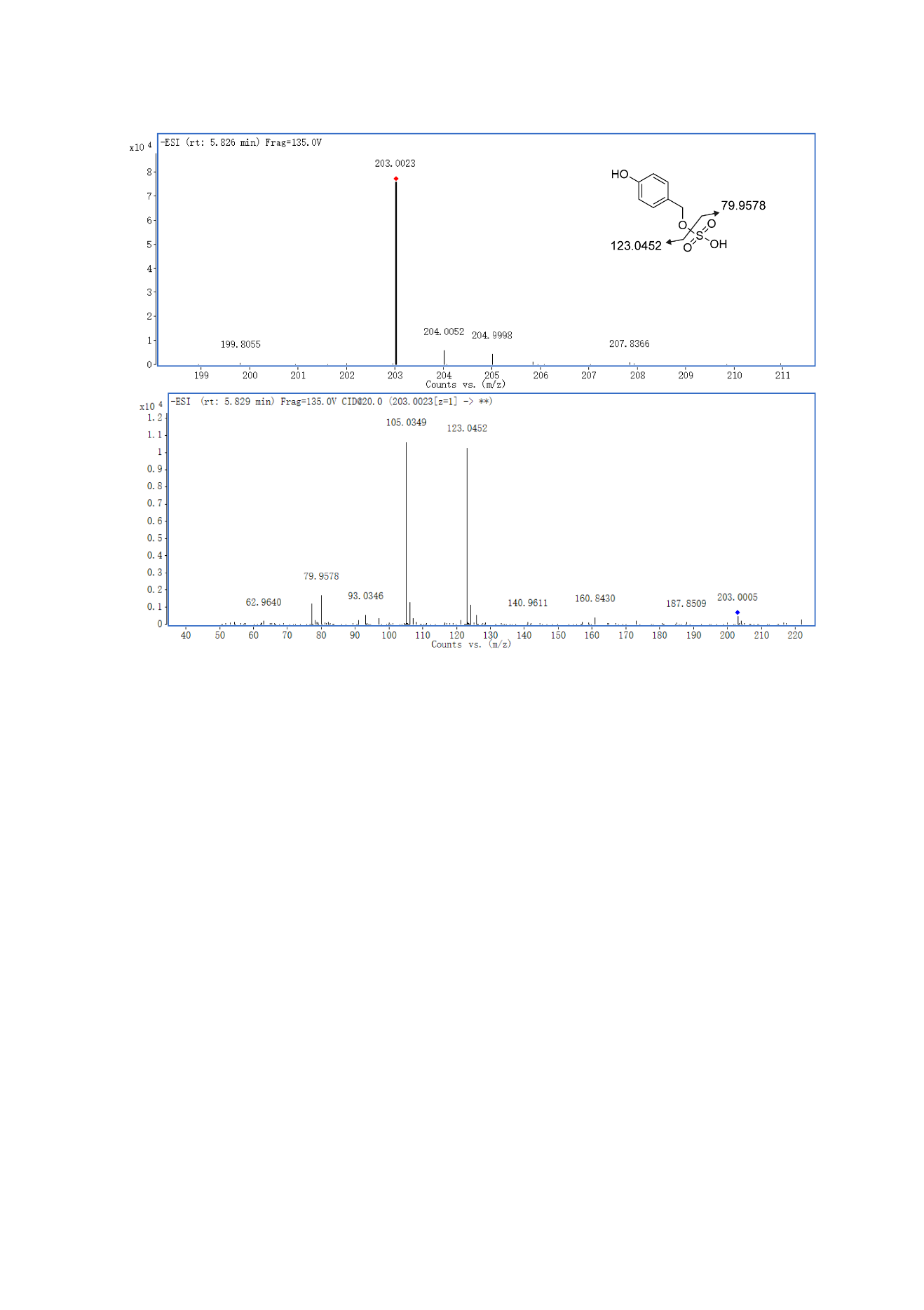


M3, eluting at 5.826 min, exhibited a [M - H]^-^ ion at *m/z* 203.0023. The fragment ions at *m/z* 123.0452 and 105.0349 indicated the presence of a HBA moiety. A mass difference of 79.9578 Da (theoretical SO_3_ loss: 79.9568 Da) further supported the incorporation of a sulfate group. Based on this evidence, M3 was identified as the sulfate conjugate of HBA, likely formed through sulfation of its hydroxyl group.

**Supplementary Figure 7**. MS and MS/MS spectra of metabolite (M4) in negative ESI mode and the proposed fragmentation pathway.


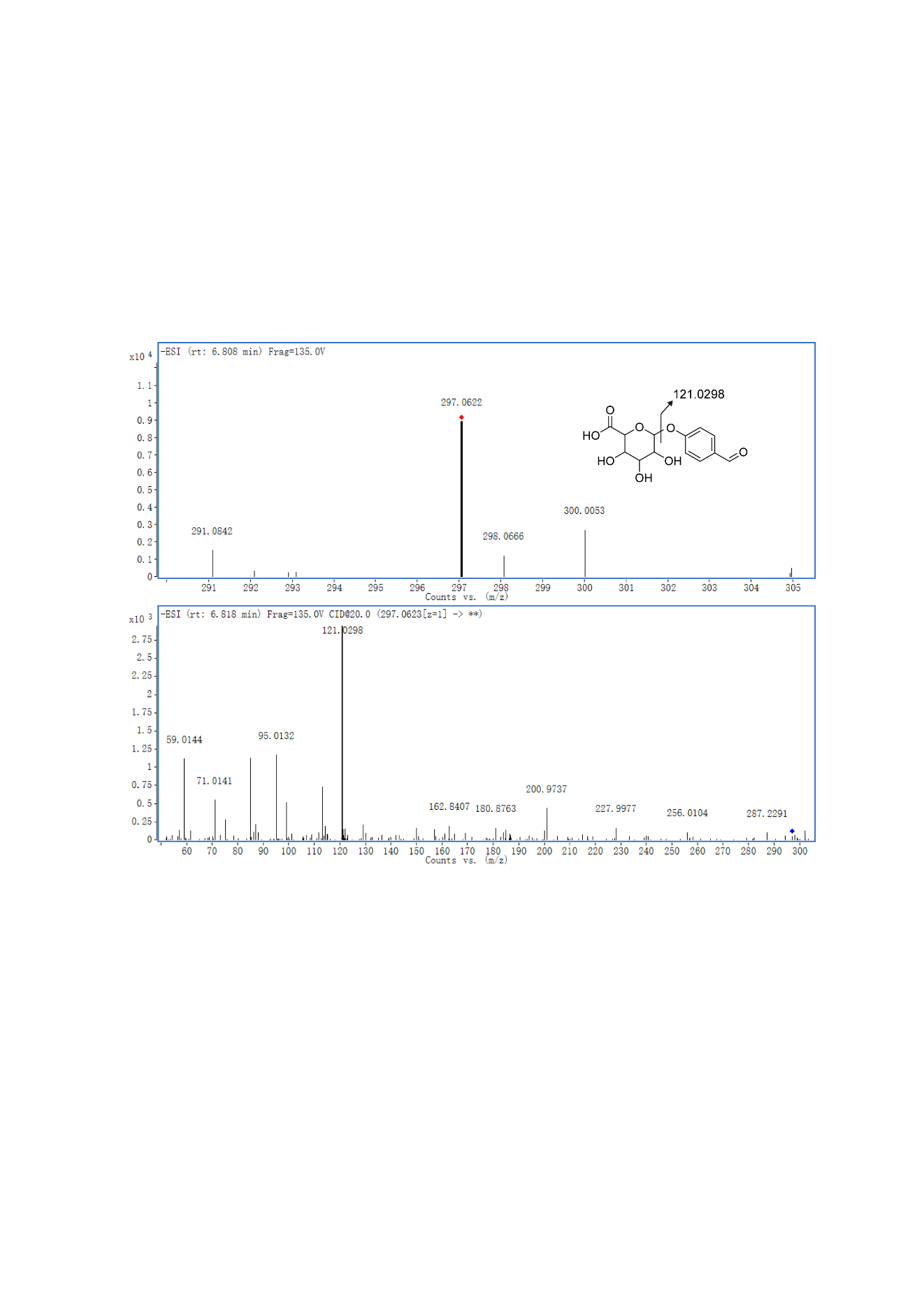


M4, eluting at 6.808 min, exhibited a [M - H]^-^ precursor ion at *m/z* 297.0622. In the MS^2^ spectrum, the product ion at *m/z* 121.0298 was characteristic of a *p*-hydroxybenzaldehyde moiety. A mass difference of 176.0324 Da (theoretical glucuronide loss: 176.0321 Da) between the precursor ion and its Phase I metabolite confirmed the presence of a glucuronide group. Based on this evidence, M4 was identified as the glucuronide conjugate of *p*-hydroxybenzaldehyde.

**Supplementary Figure 8**. MS and MS/MS spectra of metabolite (M5) in negative ESI mode and the proposed fragmentation pathway.


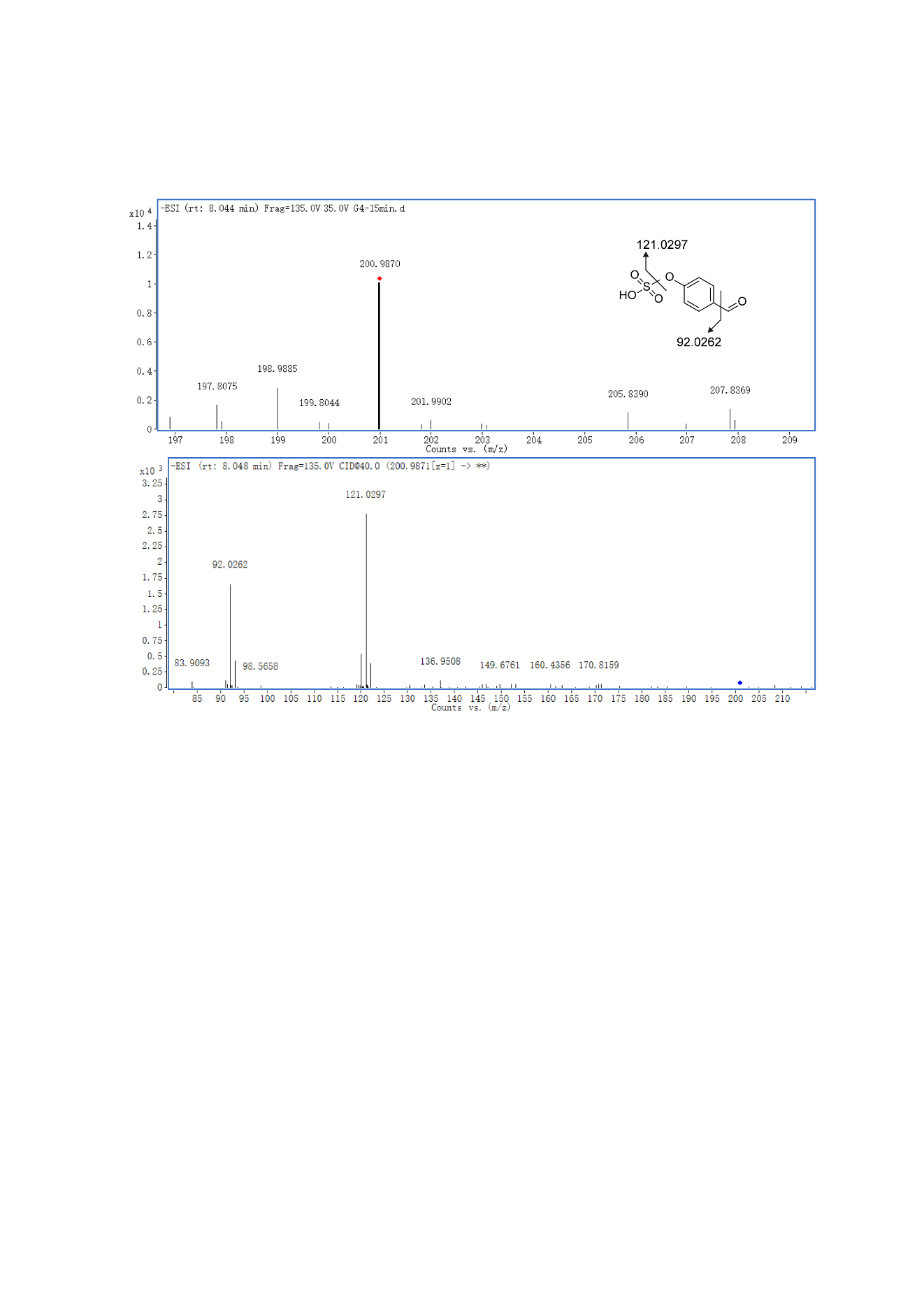


M5, eluting at 8.044 min, exhibited a [M - H]^-^ precursor ion at *m/z* 200.9870. It was identified as the sulfate conjugate of *p*-hydroxybenzaldehyde based on its diagnostic fragmentation pattern. Sequential neutral losses of SO₃ (observed Δ*m/z* −79.9573, theoretical −79.9568 Da) and HCO (observed Δ*m/z* −29.0035, theoretical −29.0027 Da) were observed: [M - H]^-^ (*m/z* 200.9870) → *m/z* 121.0297 → *m/z* 92.0262. This cleavage pathway is characteristic of sulfated phenolic aldehydes, definitively confirming the structure of M5.

**Supplementary Figure 9 and 10**. MS and MS/MS spectra of metabolite (M6-7) in negative ESI mode and the proposed fragmentation pathway.


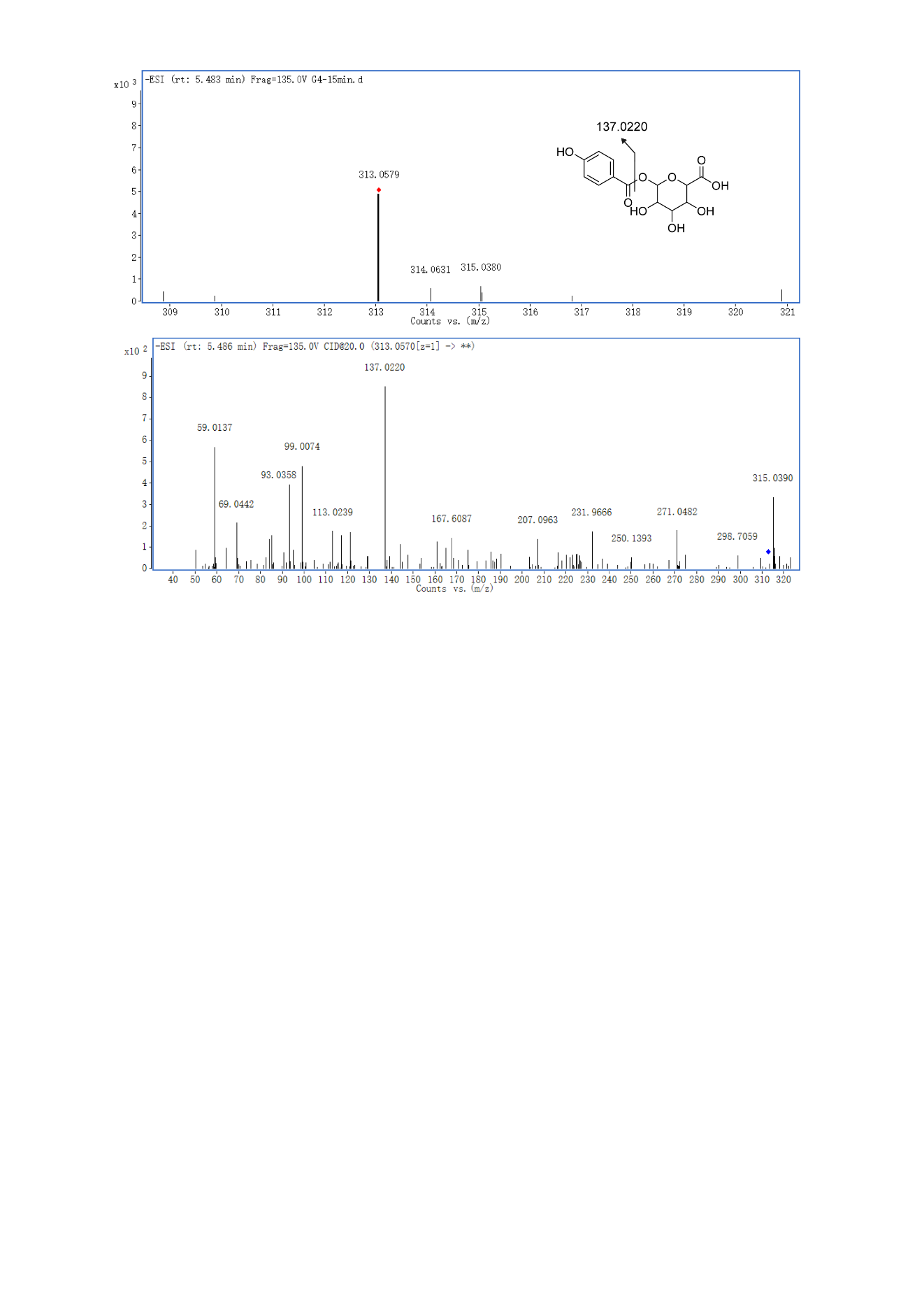

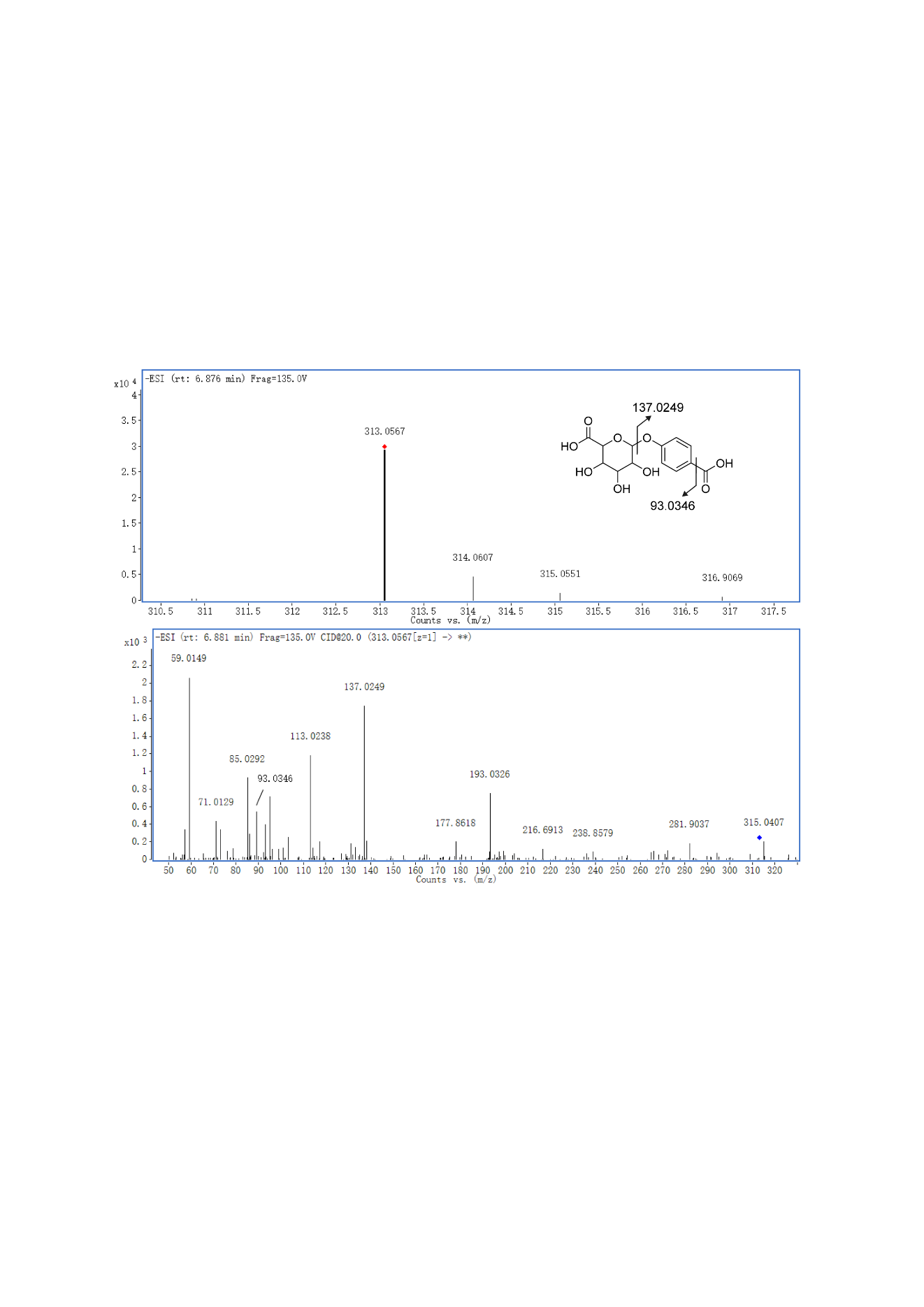


M6 (retention time: 5.483 min) and M7 (retention time: 6.876 min) shared an identical [M - H]^-^ precursor ion at *m/z* 313.057. Their MS^2^ spectra exhibited nearly identical fragment ions, including diagnostic signals at *m/z* 137.02 and 93.03, which matched the *p*-hydroxybenzoic acid moiety. The consistent mass difference of 176.03 Da (theoretical glucuronide group: 176.0321 Da) between the precursor ion (*m/z* 313.057) and the aglycone (*m/z* 137.02) confirmed glucuronide conjugation. Based on these data, M6 and M7 were tentatively characterized as glucuronide conjugates of *p*-hydroxybenzoic acid, potentially differing in the position of glucuronide attachment.

**Supplementary Figure 11**. MS and MS/MS spectra of metabolite (M8) in negative ESI mode and the proposed fragmentation pathway.


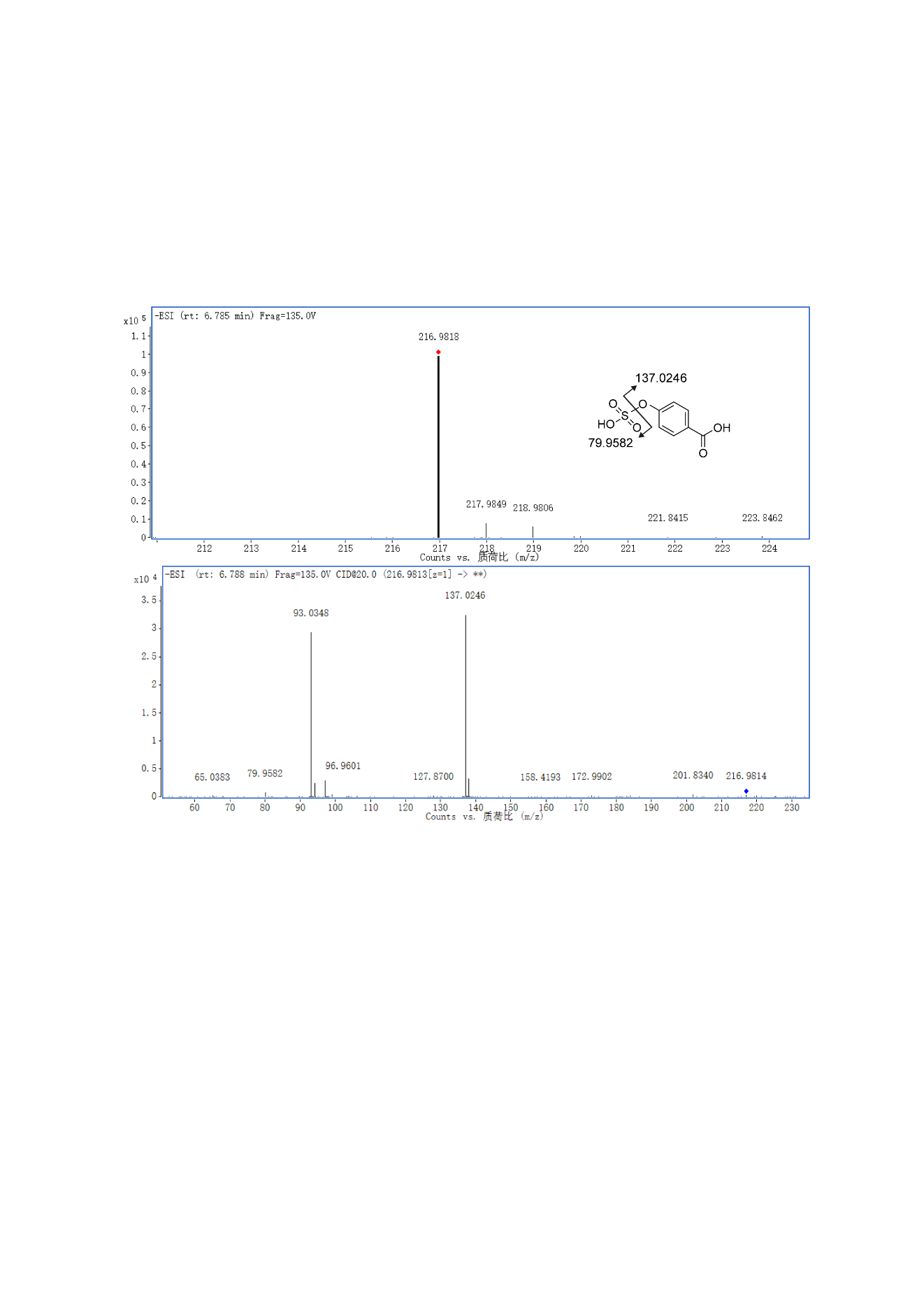


M8, eluting at 6.785 min, exhibited a [M - H]^-^ precursor ion at *m/z* 216.9818. Its MS² spectrum displayed characteristic fragment ions at *m/z* 137.0246, 93.0348, and 79.9582. The sequential neutral losses were attributed to SO₃ (observed Δ*m/z* −79.9572, theoretical −79.9568 Da; *m/z* 216.9818 → 137.0249) and COOH (observed Δ*m/z* −43.9898, theoretical −43.9898 Da; *m/z* 137.0246 → 93.0348). This fragmentation pattern is consistent with sulfated aromatic acids, leading to the identification of M8 as the sulfate conjugate of *p*-hydroxybenzoic acid, where sulfation occurs on the phenolic hydroxyl group.

**Supplementary Figure 12**. MS and MS/MS spectra of metabolite (M9) in negative ESI mode and the proposed fragmentation pathway.


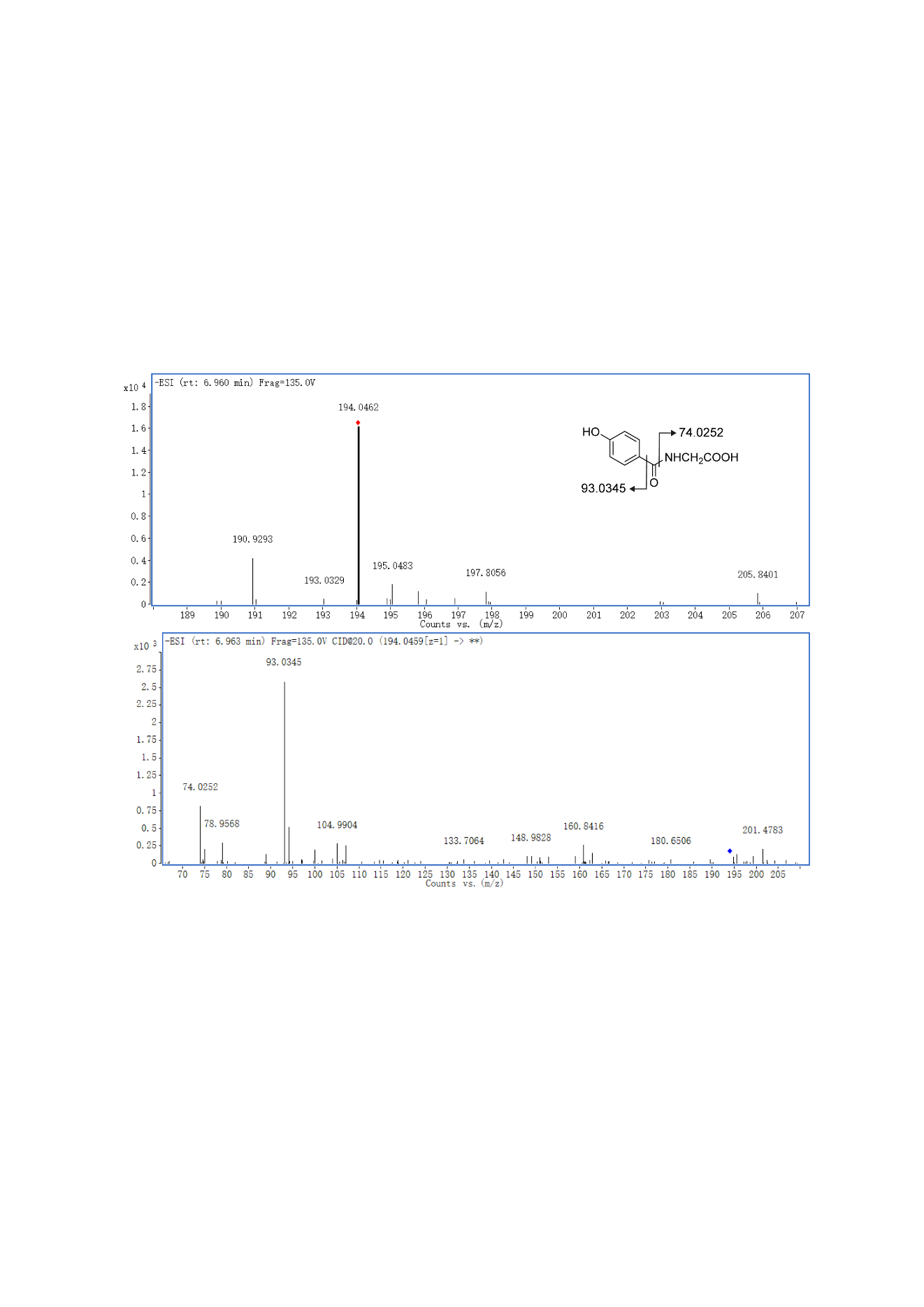


M9, eluting at 6.960 min, exhibited a [M - H]^-^ precursor ion at *m/z* 194.0462. Combined with a mass difference of 74.0252 Da (theoretical glycine conjugation: 74.0242 Da), suggested glycine conjugation (NH_2_CH_2_COO⁻), a hallmark of Phase II metabolism. The product ion a *m/z* 93.0345 in the MS^2^ spectrum matched the diagnostic fragment ion of M6–M8, confirming M9 as the Phase II metabolite formed by glycine conjugation with *p*-hydroxybenzoic acid.

**Supplementary Figure 13**. Peak areas of four metabolites M1, M2, M3, and M8 in the brain tissues of normal and MCAO rats after gavage of GEB extract.


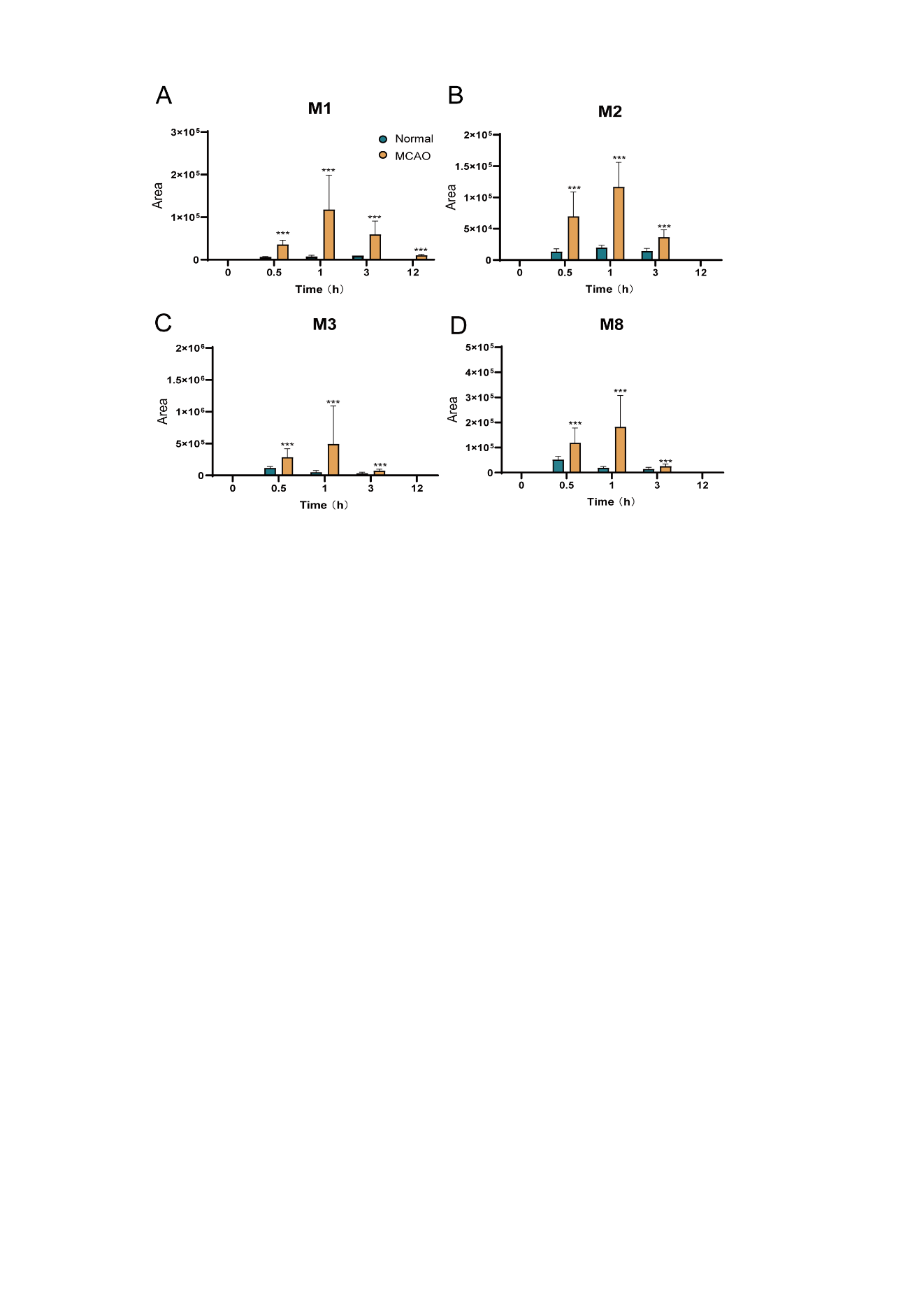


***R* code for Parishin A**

PA, formed by the dehydration-condensation of one citric acid molecule with three GAS molecules, has a large molecular weight and is highly prone to hydrolysis. Therefore, its Phase I metabolic pathways were prioritized in the analysis. Additionally, since all carboxyl groups in PA are occupied by GAS through glycosidic bonds, only the hydroxyl groups at the terminal ends of these bonds are available for Phase II metabolism. Consequently, its potential Phase II reactions are limited to glucosidation, glucuronidation, and sulfonation.

data = read_excel("text.xlsx")

x = data$Mass

a_values = 0:1

b_values = 0:1

c_values = 0:1

d_values = 0:3

e_values = 0:1

f_values = 0:1

results = data.frame(a = numeric(), b = numeric(), c = numeric(), d = numeric(), e = numeric(), f = numeric(), Mass = numeric())

for (a in a_values) {

for (b in b_values) {

if(b==1){d_values=0}

if(b==0){d_values=c(0:3)}

for (c in c_values) {

for (d in d_values) {

for (e in e_values) {

for (f in f_values) {

ParishinA = 996.3111 + a * 13.9793 + b * 15.9949 + c * 162.0528 + d * 79.9568 + e * 176.0321 + f * 162.0528

matches = which(abs((x - ParishinA) / ParishinA) < 0.00001)

if (length(matches) > 0) {

for (match in matches) {

results <- rbind(results, data.frame(a = a, b = b, c = c,d = d, e = e,f =f, Mass = x[match]))

}

}

}

}

}

}

}

}

print(results)

***R* code for Parishin B/C**

PB/PC, formed by the dehydration-condensation of one citric acid molecule with two GAS molecules, has a large molecular weight. Compared to PA, PB/PC contains an exposed carboxyl group due to the absence of one GAS moiety. This structural feature allows it to undergo Phase II metabolic reactions (e.g., glycine conjugation, glucuronidation, or sulfonation) in addition to primary Phase I metabolism (e.g., hydrolysis or oxidation). Therefore, this study systematically investigated both Phase I pathways and potential Phase II reactions based on its chemical structure.

data = read_excel("text-1.xlsx")

x = data$Mass

a_values = 0:1

b_values = 0:1

c_values = 0:2

d_values = 0:1

e_values = 0:1

f_values = 0:1

g_values = 0:2

h_values = 0:1

i_values = 0:1

j_values = 0:1

k_values = 0:1

l_values = 0:1

m_values = 0:1

n_values = 0:1

o_values = 0:1

results = data.frame(a = numeric(), b = numeric(), c = numeric(), d = numeric(),e = numeric(),f = numeric(),g = numeric(),h = numeric(),i = numeric(), j = numeric(), k = numeric(),l = numeric(),m = numeric(),n = numeric(),o = numeric(),Mass = numeric())

for (a in a_values) {

for (b in b_values) {

for (c in c_values) {

for (d in d_values)

for (e in e_values) {

if(e==1){g_values=0}

if(e==0){g_values=c(0:2)}

for (f in f_values) {

for (g in g_values) {

for (h in h_values) {

for (i in i_values) {

for (j in j_values) {

for (k in k_values) {

for (l in l_values) {

for (m in m_values) {

for (n in n_values) {

for (o in o_values) {

if(h+i+j+k+l+m+n+o>1){next}

if(a+b+c+d+e+f+g+h+i+j+k+l+m+n+o>3){next}

ParishinB = 728.2164 + a * 176.0321 + +b * 13.9793 + c * 15.9949 - d * 18.0106 + e * 162.0528 + f * 79.9568 - g * 162.0528 - h * 43.9898 + i * 57.0215 + j * 103.0092 + k* 107.0041 + l * 129.0426 + m * 144.1025 + n * 145.0198 + o* 289.0732

matches = which(abs((x - ParishinB) / ParishinB) < 0.00001)

if (length(matches) > 0) {

for (match in matches) {

results <- rbind(results, data.frame(a = a, b = b, c = c,d = d, e = e, f = f, g = g, h = h,i = i, j = j, k = k, l = l, m = m, n = n, o = o , Mass = x[match]))

}

}

}

}

}

}

}

}

}

}

}

}

}

}

}

}

print(results)

***R* code for Parishin E**

Similar to PB/PC, PE/PG（While compound PG is structurally similar to compound PE, their GAS molecules bind to different sites on citric acid.） is formed through dehydration-condensation of one citric acid molecule with one GAS molecule. With two free carboxyl groups in its structure (compared to one in PB/PC), PE is likely more susceptible to Phase II metabolism. Therefore, while analyzing its potential Phase I metabolic reactions (e.g., hydrolysis and oxidation), this study also systematically investigated all possible Phase II metabolic reactions parallel to those of PB/PC, including glycine conjugation, glucuronidation, and sulfonation.

data = read_excel("text.xlsx")

x = data$Mass

a_values = 0:1

b_values = 0:1

c_values = 0:2

d_values = 0:1

e_values = 0:1

f_values = 0:1

g_values = 0:1

h_values = 0:1

i_values = 0:1

j_values = 0:1

k_values = 0:1

l_values = 0:1

m_values = 0:1

n_values = 0:1

o_values = 0:1 results = data.frame(a = numeric(), b = numeric(), c = numeric(), d = numeric(),e = numeric(),f = numeric(),g = numeric(),h = numeric(),i = numeric(), j = numeric(), k = numeric(),l = numeric(),m = numeric(),n = numeric(),o = numeric(),Mass = numeric())

for (a in a_values) {

for (b in b_values) {

for (c in c_values) {

for (d in c_values) {

for (e in e_values) {

if(e==1){g_values=0}

if(e==0){g_values=c(0:1)}

for (f in f_values) {

for (g in g_values) {

for (h in h_values) {

for (i in i_values) {

for (j in j_values) {

for (k in k_values) {

for (l in l_values) {

for (m in m_values) {

for (n in n_values) {

for (o in o_values) {

for (p in p_values) {

if(d+i+j+k+l+m+n+o>1){next}

if(a+b+c+d+e+f+g+h+i+j+k+l+m+n+o>3){next}

ParishinE = 460.1217 + a * 176.0321 + b * 13.9793 + c * 15.9949 - d * 18.0106 + e * 162.0528 + f * 79.9568 - g * 162.0528 - h * 43.9898 + i* 57.0215 + j * 103.0092 + k * 107.0041 + l * 129.0426 + m * 144.1025 + n * 145.0198 + o * 289.0732

matches = which(abs((x - ParishinE) / ParishinE) < 0.00001)

if (length(matches) > 0) {

for (match in matches) {

results <- rbind(results, data.frame(a = a, b = b, c = c,d = d, e = e, f = f, g = g, h = h,i = i, j = j, k = k, l = l, m = m, n = n, o = o, Mass = x[match]))

}

}

}

}

}

}

}

}

}

}

}

}

}

}

}

}

}

}

print(results)

***R* code for GAS**

GAS is formed by the glycosidic linkage between HBA and glucose, and lacks free carboxyl groups in its structure. However, studies have shown that the hydroxymethyl group (-CH₂OH) of GAS can be oxidized to an aldehyde (-CHO) or carboxyl group (-COOH) during Phase I metabolism. Consequently, most of its Phase II metabolic reactions (e.g., glucuronidation or sulfation) would occur based on these oxidized derivatives, particularly the carboxylated form.

data = read_excel("text.xlsx")

x = data$Mass

a_values = 0:1

b_values = 0:1

c_values = 0:2

d_values = 0:2

e_values = 0:1

f_values = 0:1

g_values = 0:1

h_values = 0:1

i_values = 0:1

j_values = 0:1

k_values = 0:1

l_values = 0:1

m_values = 0:1

results = data.frame(a = numeric(), b = numeric(), c = numeric(), d = numeric(),e = numeric(),f = numeric(),g = numeric(),h = numeric(),i = numeric(), j = numeric(), k = numeric(),l = numeric(),m = numeric(),Mass = numeric())

for (a in a_values) {

for (b in b_values) {

for (c in c_values) {

for (d in d_values) {

for (e in e_values) {

for (f in f_values) {

for (g in g_values) {

for (h in h_values) {

for (i in i_values) {

for (j in j_values) {

for (k in k_values) {

for (l in l_values) {

for (m in m_values) {

if(g+h+i+j+k+l+m>1){next}

if(b+c>2){next}

if(a+b+c+d+e+f+g+h+i+j+k+l+m >3){next}

Gastrodin = 286.1053 + a * 176.0321 - b * 2.0157 + c * 13.9793 + d * 15.9949 + e * 162.0528 + f * 79.9568 + g * 57.0215 + h * 103.0092 + i * 107.0041 + j * 129.0426 + k * 144.1025 + l * 145.0198 + m * 289.0732

matches = which(abs((x - Gastrodin) / Gastrodin) < 0.00001)

if (length(matches) > 0) {

for (match in matches) {

results <- rbind(results, data.frame(a = a, b = b, c = c,d = d, e = e, f = f, g = g, h = h,i = i, j = j, k = k, l = l, m = m, Mass = x[match]))

}

}

}

}

}

}

}

}

}

}

}

}

}

}

}

print(results)

***R* code for HBA**

For HBA, Phase I metabolism primarily involves hydroxylation of the aromatic ring (e.g., addition of hydroxyl groups at ortho or para positions). Additionally, oxidation of its hydroxymethyl group (-CH₂OH) during Phase I metabolism may generate a carboxylic acid group (-COOH), which can subsequently undergo Phase II metabolic reactions (e.g., glucuronidation or sulfation) due to its enhanced polarity. Therefore, the metabolic pathway originating from hydroxymethyl oxidation warrants particular attention.

data = read_excel("text-1.xlsx")

x = data$Mass

a_values = 0:1

b_values = 0:1

c_values = 0:1

d_values = 0:2

e_values = 0:1

f_values = 0:1

g_values = 0:1

h_values = 0:1

i_values = 0:1

j_values = 0:1

k_values = 0:1

l_values = 0:1

results = data.frame(a = numeric(), b = numeric(), c = numeric(), d = numeric(),e = numeric(),f = numeric(),g = numeric(),h = numeric(),i = numeric(), j = numeric(), k = numeric(),l = numeric(), Mass = numeric())

for (a in a_values) {

for (b in b_values) {

for (c in c_values) {

for (d in d_values) {

for (e in e_values) {

for (f in f_values) {

for (g in g_values) {

for (h in h_values) {

for (i in i_values) {

for (j in j_values) {

for (k in k_values) {

for (l in l_values) {

if(f+g+h+i+j+k+l>1){next}

if(b+c>1){next}

if(a+b+c+d+e+f+g+h+i+j+k+l >3){next}

hydroxybenzenemethanol = 124.0524 + a * 176.0321 - b * 2.0157 + c * 13.9793 + d * 15.9949 + e * 79.9568 + f * 57.0215 + g * 103.0092 + h * 107.0041 + i * 129.0426 + j * 144.1025 + k * 145.0198 + l * 289.0732

matches = which(abs((x - hydroxybenzenemethanol) / hydroxybenzenemethanol) < 0.00001)

if (length(matches) > 0) {

for (match in matches) {

results <- rbind(results, data.frame(a = a, b = b, c = c,d = d, e = e, f = f, g = g, h = h,i = i, j = j, k = k, l = l, Mass = x[match]))

}

}

}

}

}

}

}

}

}

}

}

}

}

}

print(results)
